# Small Extracellular Vesicles from Inflamed Adipose Derived Stromal Cells Enhance the NF-*κ*B-Dependent Inflammatory/Catabolic Environment of Osteoarthritis

**DOI:** 10.1155/2022/9376338

**Published:** 2022-07-18

**Authors:** Carola Cavallo, Giulia Merli, Nicoletta Zini, Stefania D'Adamo, Luca Cattini, Michele Guescini, Brunella Grigolo, Alessandro Di Martino, Spartaco Santi, Rosa Maria Borzì, Giuseppe Filardo

**Affiliations:** ^1^Laboratorio RAMSES, IRCCS Istituto Ortopedico Rizzoli, Bologna 40136, Italy; ^2^Applied and Translational Research Center (ATRc), IRCCS Istituto Ortopedico Rizzoli, Bologna 40136, Italy; ^3^CNR Institute of Molecular Genetics “Luigi Luca Cavalli-Sforza”, Unit of Bologna, Bologna 40136, Italy; ^4^IRCCS Istituto Ortopedico Rizzoli, Bologna 40136, Italy; ^5^Department of Medical and Surgical Sciences, University of Bologna, Bologna 40138, Italy; ^6^Laboratorio di Immunoreumatologia e Rigenerazione Tissutale, IRCCS Istituto Ortopedico Rizzoli, Bologna 40136, Italy; ^7^Department of Biomolecular Science, University of Urbino Carlo Bo, Urbino 61029, Italy; ^8^Clinica Ortopedica e Traumatologica 2, IRCCS Istituto Ortopedico Rizzoli, Bologna 40136, Italy

## Abstract

The last decade has seen exponentially growing efforts to exploit the effects of adipose derived stromal cells (ADSC) in the treatment of a wide range of chronic degenerative diseases, including osteoarthritis (OA), the most prevalent joint disorder. In the perspective of developing a cell-free advanced therapy medicinal product, a focus has been recently addressed to the ADSC secretome that lends itself to an allogeneic use and can be further dissected for the selective purification of small extracellular vesicles (sEVs). sEVs can act as “biological drug carriers” to transfer information that mirror the pathophysiology of the providing cells. This is important in the clinical perspective where many OA patients are also affected by the metabolic syndrome (MetS). ADSC from MetS OA patients are dysfunctional and “inflammatory” primed within the adipose tissue. To mimic this condition, we exposed ADSC to IL-1*β*, and then we investigated the effects of the isolated sEVs on chondrocytes and synoviocytes, either cultured separately or in co-culture, to tease out the effects of these “IL-1*β* primed sEVs” on gene and protein expression of major inflammatory and catabolic OA markers. In comparison with sEVs isolated from unstimulated ADSC, the IL-1*β* primed sEVs were able to propagate NF-*κ*B activation in bystander joint cells. The effects were more prominent on synoviocytes, possibly because of a higher expression of binding molecules such as CD44. These findings call upon a careful characterization of the “inflammatory fingerprint” of ADSC to avoid the transfer of an unwanted message as well as the development of *in vitro* “preconditioning” strategies able to rescue the antiinflammatory/anticatabolic potential of ADSC-derived sEVs.

## 1. Introduction

Adipose derived stromal cells (ADSC) are an emerging therapeutic strategy for the management of osteoarthritis (OA), with promising results in terms of articular surface protection and synovial inflammation modulation in the animal model, as well as pain and functional improvement in the clinical practice [1, 2]. Adipose tissue is a convenient source for the recovery of stromal cells that can be collected much more efficiently compared to bone marrow and with a minimally invasive procedure [3]. To further exploit ADSC potential, while taking advantage of a more regulatory compliant protocol, research is increasingly focusing on their secretome [4]. Recently, a well characterized fraction of extracellular vesicles (small extracellular vesicles, sEVs) demonstrated potential disease-modifying properties by affecting the OA process. In particular, sEVs obtained from ADSC secretome by a combined protocol of precipitation/size exclusion chromatography efficiently inhibited NF-*κ*B signaling in both chondrocytes and synoviocytes [5]. Given the pivotal role of NF-*κ*B in OA development [6], ADSC-derived sEVs show a promise for the development of an effective tool to manage OA. However, a possible interaction between the adipose tissue and OA should not be overlooked. In fact, OA disproportionally affects individuals with obesity due to the complex interactions among the metabolic, biomechanical, and inflammatory factors that accompany increased adiposity [7]. These findings indicate that adipose tissue itself plays a critical role in the pathophysiology of OA.

The white adipose tissue (WAT) is a dynamic endocrine organ with major roles in the control of energy, metabolism, and immune function [8]. In obesity, adipocyte hypertrophy is responsible for the dysregulation of their secretory pattern with the onset of a status of chronic low-grade inflammation and systemic metabolic deregulation [8]. The metabolic syndrome (MetS) is a global epidemic and anticipates the age of OA onset, suggesting a distinct pathogenesis for MetOA compared to posttraumatic, ageing, genetic, or crystal OA [9]. In these dysregulated settings, adipocytes actively secrete proinflammatory cytokines [10, 11], and cells of the stromal vascular fraction including ADSC [12] even more contribute in enhancing inflammatory loops, switching their behavior from antiinflammatory to proinflammatory [12, 13]. In the niche, a shift of M2 to M1 phenotype of adipose tissue macrophages also occurs [14]. Moreover, ADSC from obese patients exhibit higher activation of caspase 3 and higher susceptibility to DNA damage [15] that is a NF-*κ*B activating event [16].

The systemic low-grade inflammation impacts on the function of major homeostatic mechanisms in joint tissues [17], thus promoting OA pathogenesis. Altogether, this puts into question the potential of ADSC and ADSC secretome from the large percentage of OA patients also affected by MetS. These pathophysiological crosstalks have been reproduced in the obese mouse model and provided evidence that interleukin 1 (IL-1) pathway and granzyme A signaling are activated [18] in adipose tissue of obese mice. IL-1 is also a pivotal cytokine in OA [19], as confirmed by functional genomics in the murine surgical OA model of the destabilization of the medial meniscus [20]. Therefore, we chose IL-1*β* to reproduce *in vitro* the inflammatory priming that ADSC receive in the adipose tissue of obese patients.

Recent evidence indicates that prior *in vitro* preconditioning of ADSC is effective in modulating their paracrine activity. It has been stated that “rather than being a constant mixture of molecular factors, MSC's secretome is known to be dependent on the diverse stimuli present in the microenvironment that MSCs encounter. As such, the composition of the MSCs' secretome can be modulated by preconditioning the MSCs during *in vitro* culture” [21]. The power of *in vitro* preconditioning of MSCs has been previously stated [22, 23] as well as the need to characterize how it affects their secretory profile. Therefore, to tease out the molecular background of the dual potential of ADSC derived from either lean or obese adipose tissue, sEVs obtained from the same ADSC used in [5] were subjected to IL-1*β* exposure, to mimic the behavior of stromal cells derived from the adipose tissue of patients with MetS. As reviewed in [24], previous data were reported pointing at the ability of IL-1*β* preconditioning of MSCs to increase their release of immunomodulatory molecules and growth factors, but few research has been addressed to the analysis of the sEVs component of their secretome [22, 23] or its effects on joint cells.

The aim of this study was therefore to identify the effects of ADSC “inflammatory” primed secretome on articular cells, with a particular focus on the effects of sEVs at both the gene and protein level on chondrocytes and synoviocytes from OA joints either cultured in monoculture or in co-culture. The latter settings may be considered an *in vitro* model of joint OA established with primary cells cultured at high density, thus respecting both the crosstalk between chondrocytes and synoviocytes and their phenotypes.

## 2. Materials and Methods

Data obtained in this work were compared to the results of our previous study investigating the antiinflammatory/anticatabolic effects of sEVs from ADSC kept in control conditions on both chondrocytes and synoviocytes following exposure of the latter cells to IL-1*β* [5]. In the present paper, we undertook a complementary evaluation of the proinflammatory/procatabolic effects of sEVs from ADSC exposed to IL-1*β* as detailed below (sEVs_IL-1) on chondrocytes and synoviocytes kept in control conditions. Parallel cultures were also exposed to sEVs from unstimulated ADSC as control. Both sEVs and sEVs_IL-1 derived from the same ADSC, the chondrocyte/synoviocyte cultures were the same, and the description of the methods partly reproduces their wording. Essential information about the enrolled OA patients that yielded the chondrocytes, the synoviocytes, and the ADSC, as well as their differential preconditioning in [5] and in the present manuscript, is reported in the Supplementary Material.

### 2.1. Adipose Tissue–Derived Stromal Cells

ADSC were isolated from three donors (two men with AGE/BMI: 35/26 and 70/28.41 and one woman, with AGE/BMI 69/43.71), starting from residual microfractured adipose tissue collected from the lower abdomen in order to be used for regenerative therapies of the knee. This tissue was processed essentially as described in [25]. ADSC were obtained from tissue that was left unutilized after regenerative medicine treatments for knee OA and therefore destined for disposal. The study was conducted in accordance with the 1975 Declaration of Helsinki. Samples were obtained after informed consent from all patients, according to the protocol detailed in the study “ADIPO_CELL” (Prot.gen.n.ro 0009545, 3 October 2017) approved by the Ethics Committee of the Rizzoli Orthopaedic Institute, Bologna, Italy.

Adipose tissue samples were washed with phosphate-buffered saline (PBS). Successively, ADSC were isolated by enzymatic digestion with 0.05% type I collagenase (Sigma-Aldrich, St. Louis, MO) at 37 °C for 1 h and filtered through a 100 *μ*m cell strainer (BD Biosciences, Bedford, MA, USA). Afterwards, cells were washed with *α*-MEM (Sigma-Aldrich) containing 15% FBS, seeded into culture flasks (20 × 10^3^ cells/cm^2^) and incubated at 37 °C in a 5% CO_2_ atmosphere in *α*-MEM with 15% FBS. At confluence, cells were detached by trypsin-EDTA treatment. Cells were seeded in *α*-MEM containing 15% of exosome-depleted FBS (Gibco, Life Technologies, Grand Island, NY, USA), cultured at 37 °C in 5% CO_2_ and continuously stimulated with 2 ng/mL of IL-1*β* (R&D systems, Minneapolis, USA), from passage 2 to passage 4. Conditioned medium (CM) of IL-1*β* treated ADSC cultures was therefore collected from passage 2 to passage 4 every third day, pooled, centrifuged, and stored in sterile conditions at −80 °C until use. To assess occurrence of IL-1*β*–induced cell senescence, ADSC of one of the patients either in control or IL-1*β*-stimulated conditions from passage 2 to passage 4 were dedicated to senescence detection. At the end, cells were recovered by trypsin. Some cells were dedicated for western blot analysis of p21. Some other cells were fixed with 4% PFA, cytospun and stained according to the Senescent Cells Histochemical Staining Kit (Sigma, USA) as previously detailed in [26]. Some other cells were dedicated for analysis of cell size by flow cytometry assessment of forward side scatter. Some other cells were placed in chamber slides and used to assess morphological changes of cell shapes and nuclei following IL-1*β* exposure.

### 2.2. sEVs_IL-1 Isolation

Purified sEVs_IL-1 were obtained from the CM of three different ADSC cultures using a protocol that combines precipitation/centrifugation and size exclusion chromatography following the manufacturer's instructions (Exo-spin, Cell Guidance Systems) to improve recovery and specificity [27]. Briefly, CMs were spun at 300×*g* for 10 min at 4 °C and then the obtained supernatant at 16000×*g* for 30 minutes at 4 °C to remove cells and cellular debris. Afterwards, supernatants were incubated overnight at 4 °C with Exo-spin Buffer (Cell Guidance Systems). After a centrifugation at 16000×*g* for 1 hour at 4 °C, the sEVs_IL-1 containing pellet was resuspended in PBS, applied to the top of an Exo-spin column, and centrifuged for 60 seconds at 50×*g*. Then, a 200 *μ*l PBS volume and a further centrifugation for 60 seconds at 50×*g* were used to elute the sEVs_IL-1 that were stored at -80 °C until use.

### 2.3. sEVs_IL-1 Quantification and Surface Epitope Characterization

sEVs_IL-1 isolated from the CM were quantified using a NanoOrange Protein Quantification Kit (Life Technologies). Briefly, sEVs_IL-1 were diluted 1 : 100 in the 1x NanoOrange working solution and incubated for 10 minutes at 90 °C. The samples were then cooled for at least 20 minutes, and the fluorescence was read at 470ex–570em nm wavelength, using a Spectra Max Gemini plate fluorometer (Molecular Devices, Sunnyvale, CA). To characterize sEVs_IL-1 surface epitopes, 5 *μ*g of sEVs_IL-1 were analyzed using the MACSPlex Exosome Kit (Miltenyi Biotec) that allows the detection of 37 surface markers and two isotype controls. sEVs_IL-1 were incubated with MACSPlex Exosome Capture Beads and with MACSPlex Exosome Detection Reagent CD9, CD63, and CD81 for 1 hour at room temperature (RT). Then, 1 ml of MACSPlex Buffer was added to each sEVs_IL-1 containing tube and left for 15 minutes at RT. The sEVs_IL-1 bound to the Capture Beads were washed by centrifuging for 5 minutes at 3000×*g* and then resuspended in 150 *μ*L of MACSPlex Buffer. Samples were analyzed with a FACS Canto II (BD Biosciences). Surface markers were calculated subtracting the median signal intensity of each bead of the control sample from the signal intensities of the respective beads incubated with sEVs_IL-1.

### 2.4. Chondrocyte and Synoviocyte Isolation

Chondrocytes (derived from 1 man with AGE/BMI: 58/24.34 and 2 women, with AGE/BMI: 75/24.17 and 60/25.64) and synoviocytes (derived from 2 men, with AGE/BMI: 69/32.88 and 69/29.07) were isolated from the OA knees of patients undergoing joint replacement surgery. The study was conducted in accordance with the 1975 Declaration of Helsinki. Samples were obtained after informed consent from all patients, according to the protocol detailed in the study “ADIPO_CELL” (prot.gen.n.ro 0009545, 3 October 2017) approved by the Ethics Committee of the Rizzoli Orthopaedic Institute, Bologna, Italy.

Briefly, cartilage and synovial tissues were washed twice with PBS and minced into small pieces. Chondrocytes were obtained from three patients and isolated by sequential enzymatic digestions: 1 h with pronase (Sigma) and 1-2 h with 0.2% collagenase (Sigma) at 37 °C. Isolated chondrocytes were filtered by 100 *μ*m and 70 *μ*m nylon meshes, washed, and centrifuged. Cells were seeded at 8 × 10^3^ cells/cm^2^ in T150 flasks and cultured under conventional monoculture conditions in DMEM (Sigma) with 10% FCS. Chondrocytes were frozen at passage 0, collected upon confluency. Synoviocytes were isolated from the synovium of two patients. The tissue was minced into small pieces and cultured in DMEM (Sigma) with 10% FCS for two weeks to allow cell release. Synoviocytes from each patient were frozen at passage 0. When the planned number of samples was collected, stored chondrocytes and synoviocytes were thawed and seeded at 8 × 10^3^ cells/cm^2^ in T150 flasks. Once enough cells (either chondrocytes or synoviocytes) were available, they were pooled and used for the experiments as described below. Chondrocytes and synoviocytes were used at passages 2 and 3, respectively.

### 2.5. Monoculture and Co-Culture Experiments

The effects of three different purified sEVs_IL-1 preparations were evaluated on pooled chondrocytes and pooled synoviocytes in either monoculture or co-culture. Indeed, the latter represents a culture model that reproduces *in vitro* the crosstalk and positive paracrine feedback loops among the joint cells.

For monoculture experiments, chondrocytes and synoviocytes were thawed and seeded in 24-well plates at high density (2 × 10^5^/cm^2^ and of 6 × 10^4^/cm^2^, respectively) for 3 days in DMEM (Sigma) with 10% FCS. Afterwards, medium was substituted with fresh 10% FBS-DMEM containing 10 *μ*g/mL of sEVs_IL-1 (or of sEVs, as control) or without sEVs (CTR group). Co-cultures were set up by seeding 2 × 10^5^ chondrocytes in the lower chamber of a 24-well plate and 6 × 10^4^ synoviocytes in the transwell (0.4 *μ*m pore size, Corning, Toledo, OH). These cultures were at first set up separately and left to adhere and reestablish extracellular matrix for 3 days in DMEM (Sigma) with 10% FCS. The following day, the co-cultures were assembled, and the medium was substituted with fresh DMEM +10% FBS containing 10 *μ*g/mL of sEVs_IL-1 (or of sEVs, as control) or without sEVs. All evaluations were carried out at 4 and 15 hours after the addition of sEVs_IL-1(or of sEVs, as control).

### 2.6. Real-Time PCR

Cells from monocultures and co-cultures kept in either CTR or sEVs_IL-1 (or of sEVs, as control) conditions were analyzed by Real-Time RT-PCR at 4 and 15 hours to investigate the expression of IL-1*β*, IL-6, IL-8, MCP-1, TNF-*α*, IL-10, MMP-1, MMP-10, ADAMTS-4, ADAMTS-5, MMP-13, COX-2, INOS, and VEGF (Table 1). Total RNA was isolated using TRIZOL reagent (Invitrogen) following the manufacturer's recommended protocol [28]. The samples were then treated with DNase I (DNA-free Kit; Ambion, Life Technologies) and RNA quantified using Nanodrop spectrophotometer (EuroClone S.p.a.). The RNA (200 ng each) was reverse transcribed using the SuperScript Vilo cDNA synthesis Kit (Invitrogen), according to the manufacturer's protocol. Real-time PCR was run in a LightCycler Instrument (Roche Molecular Biochemicals, Indianapolis, IN) using SYBR Premix Ex Taq (Takara, Clontech Laboratories, Mountain View, CA) with the following protocol: initial activation at 95 °C for 10 minutes, amplification for 45 cycles at 95 °C for 5 seconds, and at 60 °C for 20 seconds. mRNA levels were calculated for each target gene and normalized using the reference gene GAPDH according to the formula 2^-*Δ*Ct^ [29] and expressed as a percentage of the reference gene. To check for reliability of the gene transcription data, we performed a stability test, by comparing the threshold cycles of GAPDH of the cDNA samples.

### 2.7. Quantification of Secreted Factors

The supernatants collected from monocultures and co-cultures at 4 and 15 hours were centrifuged to eliminate cellular debris and particulates and stored at -80 °C until use. Successively, samples were evaluated for the release of IL-1*β*, IL-1ra, IL-2, IL-4, IL-6, IL-8, IL-9, IL-10, IL-12, IL-13, IL-15, basic FGF, eotaxin, G-CSF, GM-CSF, IFN-*γ*, IP-10, MCP-1, MIP-1*α*, MIP-1*β*, RANTES, TNF-*α*, and VEGF using the Bio-Plex Protein Array System (Bio-Rad Laboratories, Hercules, CA and Millipore Corporation, Billerica, MA) following the manufacturer's instructions. A quality control of the calibration curve was undertaken so that only standard levels between 70% and 130% of the expected values were considered accurate and were used.

### 2.8. p65 Immunofluorescence and Imaging

Chondrocytes and synoviocytes were seeded in 8 wells chamber slides at a density of 3 × 10^4^/cm^2^ cells for 3 days in DMEM (Sigma) with 10% FCS. Afterwards, the medium was removed and substituted in half of the wells with fresh DMEM +10% FBS (CTR) and in the other half with the same medium containing 10 *μ*g/mL of sEVs_IL-1 and cultured for 4 and 15 hours. At the end of each experimental time point (4 h and 15 h), the cells were fixed with 4% PFA, treated for antigen unmasking with a solution of 0.02 U/mL of chondroitinase ABC in 50 mM TRIS-HCl pH 8 (for chondrocytes) or with a solution of 0.1% of Triton-X-100/PBS (for synoviocytes) and blocked with 4% bovine serum albumin (BSA) (Sigma-Aldrich) in 0.1% Triton-X-100/TBS (dilution buffer, used for primary and secondary antibodies dilution) to avoid unspecific bindings. Successively, cells were incubated with 15 *μ*g/mL of rabbit anti-human p65 (AbCAM AB7970, rabbit polyclonal antibody, 5 *μ*g/ml) for 4 h at RT, followed by incubation with 15 *μ*g/mL of donkey anti-rabbit IgG Alexa Fluor 555 (Thermofisher Scientific). The nuclei were labeled with a DAPI solution (Sigma). Slides were mounted with the antifade reagent and examined under the Nikon A1-R confocal laser scanning microscope.

Confocal analysis was performed using a Nikon A1 confocal laser scanning microscope, equipped with a 100×/1.49 NA objective lens and with 405 and 561 nm laser lines. Z-stacks were collected at optical resolution of 80 nm/pixel, stored at 12-bit with 4096 different grey levels, pinhole diameter set to 1 Airy unit, and z-step size to 200 nm. Confocal images were processed using Richardson-Lucy deconvolution algorithm by using NIS-Elements Advanced Research software (Nikon, Tokyo, Japan). A large area of the samples was observed, and 5 representative cells for each condition were analyzed and processed as described thereafter.

Colocalization analysis was evaluated by comparing the equivalent pixel positions of blue and red signals of fluorophores in each of the acquired images (optical sections). A two-dimensional scatter plot diagram of the individual pixels from the paired images was generated and a threshold level of signal to be included in the analysis was selected. Pixels with intensity values greater than 50% grey levels (on a scale from 0 to 4096) were selected for both signals, and the colocalization binary maps that indicate regions containing highly colocalized signals were imaged and merged (in white) to the blue and red signals [30, 31]. The colocalization of the fluorochromes was quantified using Pearson's colocalization coefficient (*r*), derived from 15 analyzed optical sections and expressed as percentage ± SD.

### 2.9. Western Blot Analysis of NF-*κ*B Activation

The tuning of NF-*κ*B signaling was further investigated by western blot analysis on both chondrocytes (100,000) and synoviocytes (60,000) plated in 24-well plates in CTR and sEVs_IL-1 conditions at both 4 and 15 hours since delivery. At the time of collection, the medium was removed, and the cells were recovered with a scraper using a small volume of cold PBS with the addition of inhibitors of phosphatases and proteases. Then, the cells were gently centrifuged and lysed with 20 *μ*l of RIPA buffer. The samples were subsequently loaded, run on the acrylamide gels, and transferred to PVDF membranes as detailed previously [5].

Western blot was carried out with the following antibodies, Phospho-NF-*κ*B p65(Ser536) (rabbit monoclonal antibody, clone 93H1, used at 1 : 1,000, Cell signaling Technology #3033) and *β*-actin (mouse monoclonal, clone AC-74, used at 0.8 *μ*g/ml Sigma # A2228) that served as loading control. Appropriate anti-species HRP conjugated secondary antibodies were from Jackson laboratories.

### 2.10. Statistical Analysis

Prior testing for normality of data distribution was carried out using the Kolgomorov Smirnov (K-S) test. However, since normality tests have little power to detect whether or not a sample comes from a Gaussian population when the sample is tiny, to increase the size of the samples, the K-S test was performed on the data combined from similar conditions (e.g., expression of a given gene in a given condition and at a given time point, cumulating the data in monoculture and co-culture).

Data were expressed as mean and standard deviation (mean ± SD) and analyzed and graphed using the GraphPad Prism 5.0 software (Graph Pad Software, La Jolla, CA, USA). Comparison among two groups (comparison of sEVs surface epitope characterization between sEVs and sEVs_ IL-1) were undertaken by mean of the Student's *t*-test for paired samples. Comparisons among multiple groups (CTR, sEVs, or sEVs_IL-1 treated cells at 4 and 15 hours) were carried out with ANOVA, followed by Tukey's post hoc test. Again, the differences were considered significant and evidenced as ∗*p* < 0.05; ∗∗*p* < 0.01; and ∗∗∗*p* < 0.001.

## 3. Results

### 3.1. sEVs_IL-1 Quantification and Characterization

At the end of the isolation procedure starting from IL-1*β* treated ADSC-CM (50 mL), a 200 *μ*l volume of purified sEVs_IL-1 was obtained. The measured protein concentration (mean ± standard deviation, *n* = 3) as evaluated with NanoOrange was 122.4 ± 9.86 *μ*g/mL per sample.

**Figure 1:**
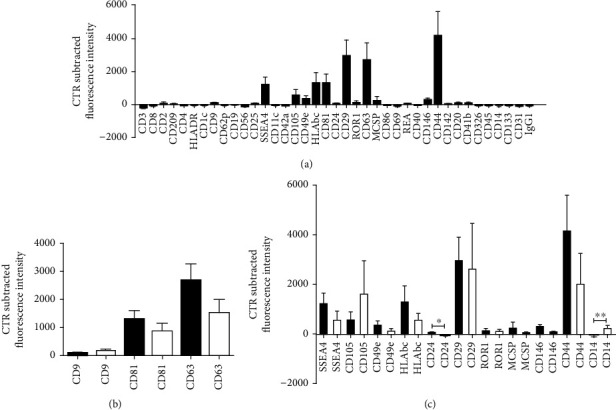
sEVs_IL-1 Surface Epitope characterization. (a) Whole MACSPlex Exosome Kit surface assessment of 37 surface markers and 2 isotype controls of different purified sEVs_IL-1 preparations (*n* = 3) from IL-1*β* treated ADSC (black graphs). Data are expressed as net fluorescence intensity (control subtracted fluorescence intensity) and displayed as mean ± *SD*. (b) Side-by-side comparison of the expression of tetraspanins (CD9, CD81, and CD63) in sEVs obtained from IL-1*β* treated ADSC (sEVs_IL-1, *n* = 3, black graphs) or untreated ADSC (sEVs, *n* = 3, white graphs). (c) Side-by-side comparison of the expression of other 11 detectable surface markers in sEVs obtained from IL-1*β* treated ADSC (sEVs_IL-1, *n* = 3, black graphs) or untreated ADSC (sEVs, *n* = 3, white graphs). The latter correspond to data presented in [5]. Since the ADSC cultures were the same, comparisons were undertaken by mean of the Student's *t*-test for paired samples and statistically significant differences evidenced as ∗*p* < 0.05 and ∗∗*p* < 0.01.

The multiplex bead-based assay coupled with a flow cytometric analysis confirmed that the sEVs_IL-1 samples were positive for the specific sEVs markers: CD63, a tetraspanin accumulating in multivesicular bodies, and CD9/CD81 that are localized mainly at the plasma membrane, even if with different intensity (Figure 1(a), reporting the whole surface characterization of sEVs_IL-1 with details of all surface markers and isotypes).

Since the ADSC used to obtain both sEVs_IL-1 (from IL-1 stimulated ADSC) and sEVs (from control ADSC) were the same, the surface characterization data were compared with those presented in [5].  Figure 1(b) and Supplementary Figure 1 shows side by side the comparison of surface characterization of tetraspanins, major sEVs markers. CD63 was the tetraspanin expressed at the highest level in both sEVs and sEVs_IL-1*β* (with CD63 expressed approximately tenfold the level of CD9 and twofold the level of CD81). These findings are in keeping with what reported by [32].

The comparison between sEVs and sEVs_IL-1 yielded only a few statistically significant differences (CD24 and CD14, the first one higher in sEVs_IL-1, and the second lower in sEVs_IL-1) but some trends of differential expression (Figure 1(c)). Moreover, sEVs_IL-1 showed a strong positivity for the mesenchymal stromal markers CD29, CD44, and CD105 and for the stage specific embryonic antigen- 4 (SSEA-4) usually associated to cellular pluripotency, as shown in Figure 1(c).

Of the 39 markers analyzed, besides the two mentioned above, 5 showed a differential (though not statistically significant) epitope fluorescent intensity, with concordant changes between sEVs_IL-1 and sEVs: SSEA4, CD49e, CD146, and CD44 expression levels were higher in sEVs_IL-1 compared to sEVs. On the contrary, CD9, CD105, and CD14 were more expressed in sEVs with respect to sEVs_IL-1 (Figure 1(c)). Notably, the comparison of the fluorescence intensity of the surface proteins performed using the cumulative data (*n* = 6) confirmed that CD44 intensity is significantly higher (∗∗∗*p* < 0.001) compared to all the other proteins but CD29 and CD63 (Supplementary Figure 1). This information is valuable taking into account the role of CD44 in sEVs binding to pericellular hyaluronan and further internalization [33]. Further characterization of sEVs at the electron microscopy and nanotracker analysis has been previously reported [5].

### 3.2. sEVs_IL-1 Effects on Gene Expression in Chondrocytes and Synoviocytes

As shown in Supplementary Figure 3, GAPDH proved to be a reliable reference gene since it showed a great stability [34] in the experimental conditions used in this and in the previous study [5].

**Figure 2:**
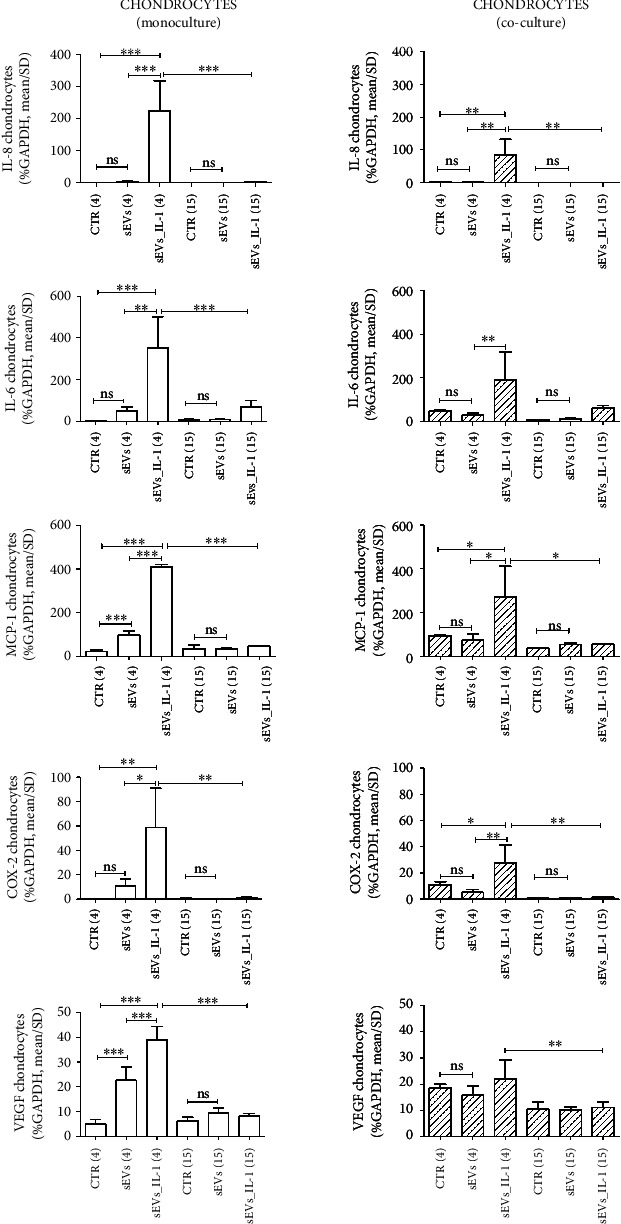
sEVs_IL-1 effects on gene expression of major inflammatory genes in chondrocytes kept in control conditions. The figure reports the effects on gene expression of major inflammatory molecules (IL-8, IL-6, and MCP-1) and molecules involved in angiogenesis (VEGF) and pain (COX-2) in chondrocytes grown in both monoculture (white pattern, left graphs) and co-cultured with synoviocytes (white dashed pattern, right graphs) in CTR, sEVs, or sEVs_IL-1 conditions. Data were normalized to GAPDH. Each graph reports data collected from 3 independent sEVs samples and expressed as means ± SD. The means of the groups were compared by ANOVA, followed by Tukey's post hoc test, with ∗*p* < 0.05, ∗∗*p* < 0.01, and ∗∗∗*p* < 0.001.

**Figure 3:**
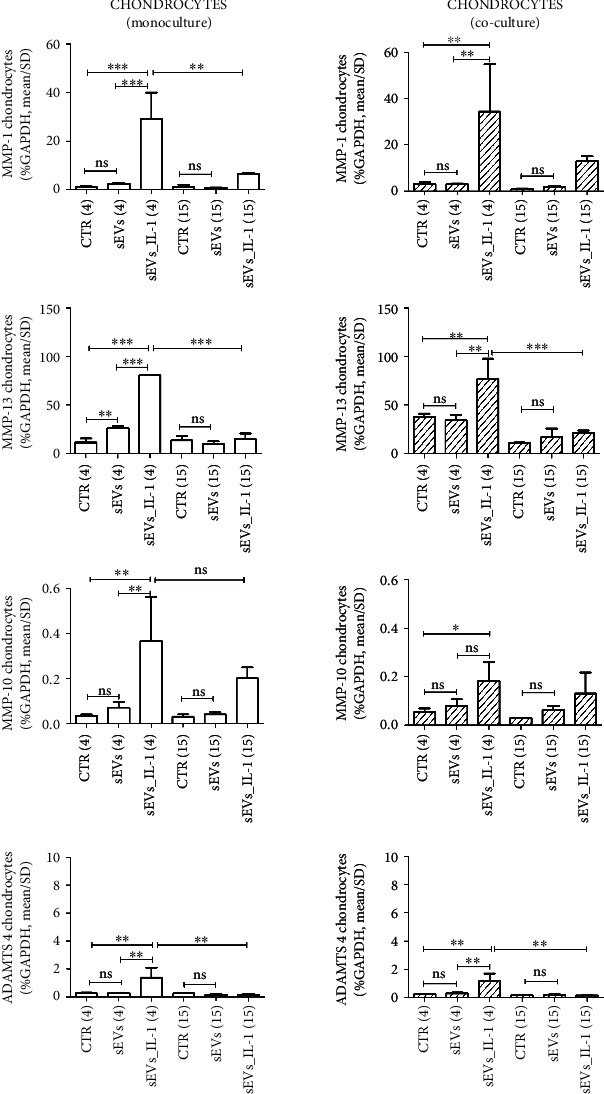
sEVs_IL-1 effects on gene expression of catabolic genes in chondrocytes kept in control conditions. The figure reports the effects on gene expression of major catabolic enzymes (MMP-1, MMP-13, MMP-10, and ADAMTS 4) in chondrocytes grown in both monoculture (white pattern, left graphs) and co-cultured with synoviocytes (white dashed pattern, right graphs) in CTR, sEVs, or sEVs_IL-1 conditions. Data were normalized to GAPDH. Each graph reports data collected from 3 independent sEVs samples and expressed as mean ± SD. The means of the groups were compared by ANOVA, followed by Tukey's post hoc test, with ∗*p* < 0.05, ∗∗*p* < 0.01, and ∗∗∗*p* < 0.001.

**Figure 4:**
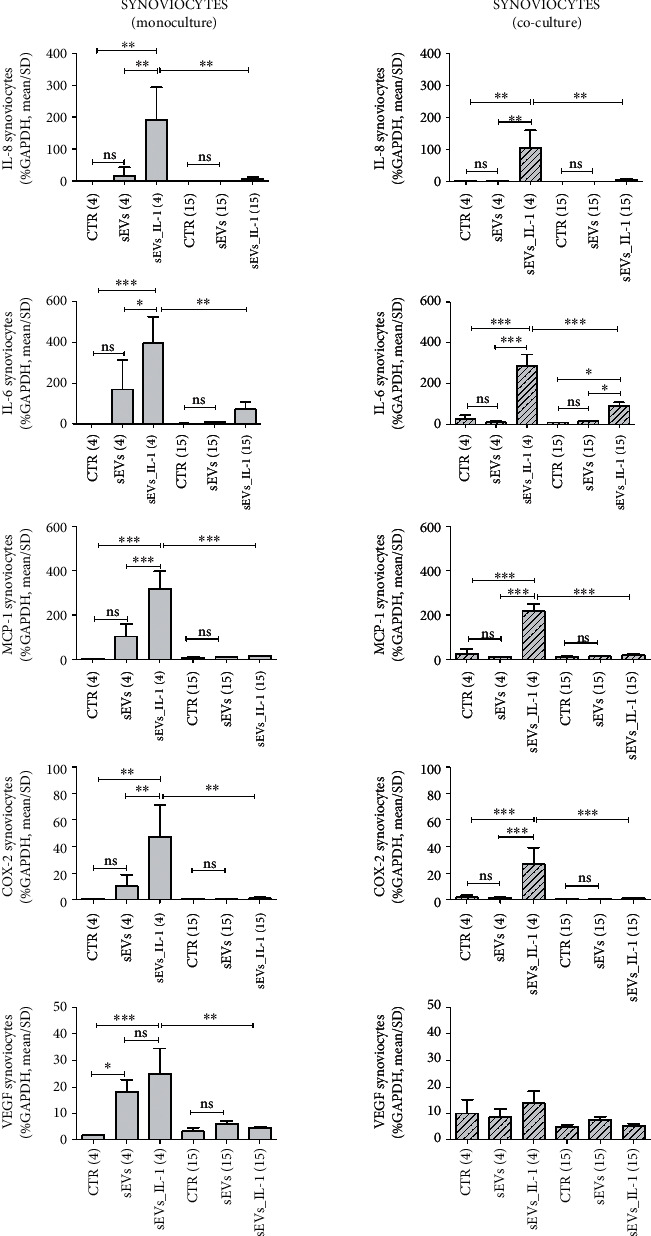
sEVs_IL-1 effects on gene expression of major inflammatory genes in synoviocytes kept in control conditions. The figure reports the effects on gene expression of major inflammatory molecules (IL-8, IL-6, and MCP-1) and molecules involved in angiogenesis (VEGF) and pain (COX-2) in synoviocytes grown in both monoculture (grey pattern, left graphs) and co-cultured with chondrocytes (grey dashed pattern, right graphs) in CTR, sEVs, or sEVs_IL-1 conditions. Data were normalized to GAPDH. Each graph reports data collected from 3 independent sEVs samples and expressed as means ± SD. The means of the groups were compared by ANOVA, followed by Tukey's post hoc test, with ∗*p* < 0.05, ∗∗*p* < 0.01, and ∗∗∗*p* < 0.001.

**Figure 5:**
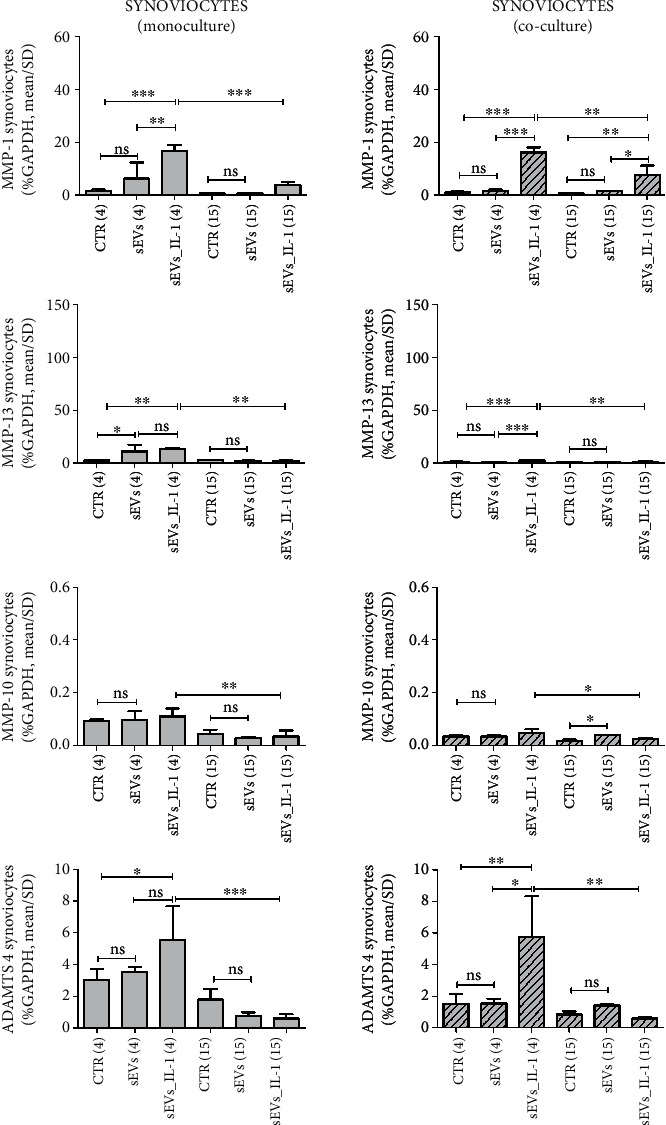
sEVs_IL-1 effects on gene expression of catabolic genes in synoviocytes kept in control conditions. The figure reports the effects on gene expression of major catabolic enzymes (MMP-1, MMP-13, MMP-10, and ADAMTS 4) in synoviocytes grown in both monoculture (grey pattern, left graphs) and co-cultured with chondrocytes (grey dashed pattern, right graphs) in CTR, sEVs, or sEVs_IL-1 conditions. Data were normalized to GAPDH. Each graph reports data collected from 3 independent sEVs samples and expressed as mean ± SD. The means of the groups were compared by ANOVA, followed by Tukey's post hoc test, with ∗*p* < 0.05, ∗∗*p* < 0.01, and ∗∗∗*p* < 0.001.

To evaluate if sEVs_IL-1 were able to upregulate the expression of catabolic and inflammatory genes in both chondrocytes and synoviocytes kept in control conditions, the expression of several genes involved in OA pathophysiology was evaluated, including inflammatory cytokines/chemokines (IL-1*β*, IL-6, IL-8, MCP-1, TNF-*α*, and IL-10), catabolic enzymes (MMP-1,-10, -13, ADAMTS-4, and -5), and molecules related to angiogenetic/pain processes such COX-2, INOS, and VEGF. The results are shown in Figures 2–5 (Figure 2: major genes involved in inflammation in chondrocytes in both monoculture and co-culture; Figure 3: major genes involved in matrix remodeling in chondrocytes in both monoculture and co-culture; Figure 4: major genes involved in inflammation in synoviocytes in both monoculture and co-culture; and Figure 5: major genes involved in matrix remodeling in synoviocytes in both monoculture and co-culture) of the main manuscript and in Supplementary material (Supplementary Figures 4 and 5).

For a clearer overview of the differential effects on the two cell types each considered in both monoculture and in co-culture, the same scale of the *y*-axis was used for each gene. In addition, in each comparison at both 4 and 15 hours, the values obtained with unstimulated sEVs (obtained from control ADSC) were shown, to tease out the specific effects of sEVs_IL-1. As shown in Figures 2–5 and Supplementary Figures 4 and 5, in most cases, the difference between CTR and sEVs (from control ADSC) was not significant, or in the case of a statistical difference the sEVs_IL-1 induced a significantly higher gene transcription compared to sEVs as seen, e.g., for chondrocytes at 4 h for MCP-1, VEGF (Figure 2), or MMP-13 (Figure 3).

In general, compared to the CTR, sEVs_IL-1 induced a different gene expression, strictly related to the experimental time point. In chondrocytes, sEVs_IL-1 significantly increased the expression of some cytokine/chemokine genes (IL-6, IL-8, and MCP-1; Figure 2), catabolic enzymes (MMP-1, -10, -13, and ADAMTS-4; Figure 3), and angiogenetic/pain factors (COX-2 and VEGF; Figure 2) with respect to the CTR at 4 h. This effect was somehow attenuated at later time points (15 hr) when the levels of some of these genes (IL-6, IL-8, MCP-1, COX-2 and VEGF, MMP-1, -13, and ADAMTS-4) were significantly reduced compared to 4 hours, suggesting that the effects of sEVs_IL-1 were rather short term and/or that reduced levels at 15 hour could be consistent with the known instability of transcripts for inflammatory molecules that harbor instability elements, as extensively reviewed in [35].

Synoviocytes showed a similar trend: at 4 h, the presence of sEVs_IL-1 induced a higher expression of cytokines/chemokines, extracellular matrix degradative enzymes, and inflammatory/pain genes (IL-6, -8, MCP-1, IL-1*β*, TNF-*α*, MMP-1, -13, ADAMTS-4, COX-2, and VEGF,), compared to the CTR (Figures 4 and 5 and Supplementary Figure 5). As with the chondrocytes, in comparison with the control cells, at 15 h, sEVs_IL-1 did not further increase the expression of these genes [36].

Moreover, the effects of sEVs_IL-1 addition on chondrocytes and synoviocytes grown in co-culture, a culture condition able to mimic the articular environment, were evaluated. In chondrocytes, sEVs_IL-1 significantly increased the expression of IL-8, MCP-1, COX-2, IL-10, INOS, MMP-1, -13, -10 and ADAMTS-4 compared to the CTR at 4 h (Figures 2–3 and Supplementary Figure 4). These effects were also short term, since compared to the 4 h time point, at 15 h, the levels of IL-8, MCP-1, VEGF, MMP-13, ADAMTS-4, INOS and COX-2 were significantly reduced.

Synoviocytes in co-culture showed a similar trend (Figures 4–5 and Supplementary Figure 5): at 4 h, a higher expression of IL-6, IL-8, MCP-1, IL-1*β*, TNF-*α*, MMP-1, -13, ADAMTS-4, and COX-2 was noticed in sEVs_IL-1 compared to the CTR. Compared to the 4 h levels, at 15 h most of these mRNA were markedly downregulated (IL-6, IL-8, MCP-1, TNF-*α*, IL-1*β*, MMP-1, -13, ADAMTS-4, and COX-2). Interestingly, compared to CTR, even in co-cultured synoviocytes, sEVs_IL-1*β* were able to significantly upregulate the expression of the same genes belonging to both the inflammatory and catabolic family. Overall, in both monoculture and co-culture, chondrocytes behaved similarly to synoviocytes with regard to genes of the inflammatory family. Concerning the genes of the catabolic family, chondrocytes appeared more active with regards to MMP-1, 13, and 10, while ADAMTS4 showed a similar trend in chondrocytes and synoviocytes, with a higher level of expression in the latter cell type.

### 3.3. sEVs_IL-1 Effects on Secreted Factors

**Figure 6:**
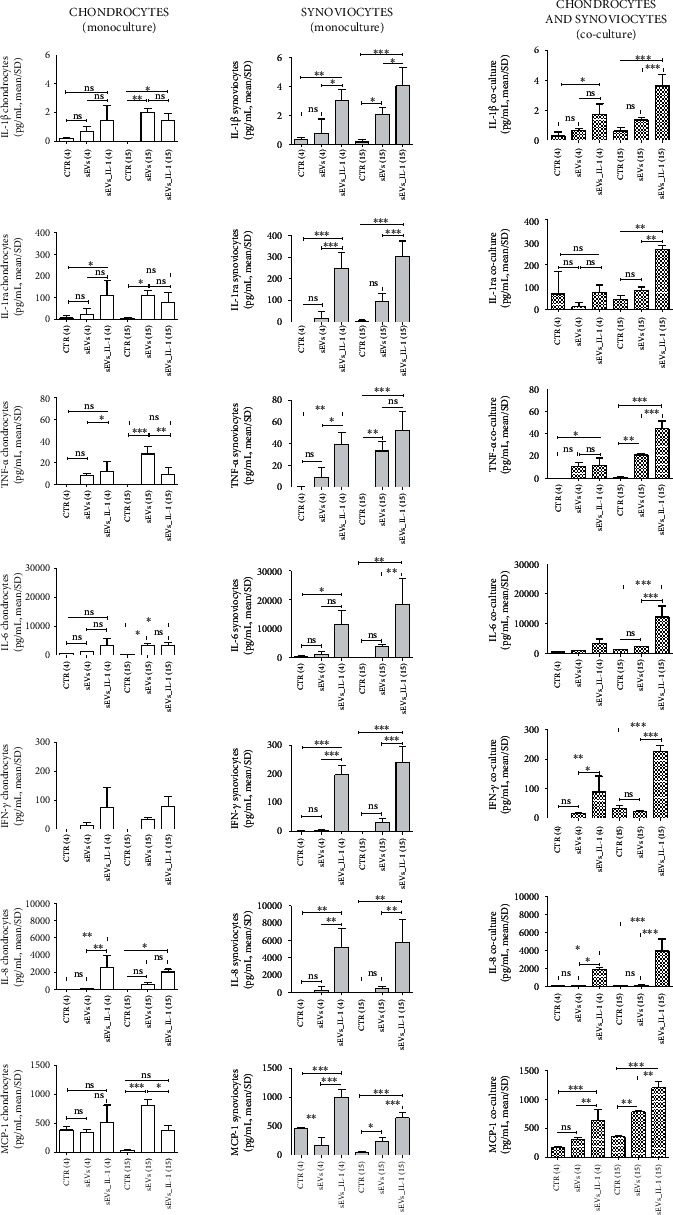
sEVs_IL-1 effects on the protein release of major inflammatory molecules (IL-1*β*, IL-1ra, TNF-*α*, IL-6, IFN-*γ*, IL-8, and MCP-1) as assessed by Bioplex. Flanked results obtained from chondrocytes (white pattern), synoviocytes (grey pattern), and co-culture of these cells (pixelated pattern). For each cell type, results are shown obtained in CTR, sEVs, or sEVs_IL-1 conditions. Each graph reports data collected from 3 independent sEVs samples and expressed as means ± SD. Data were compared by ANOVA, followed by Tukey's post hoc test, with ∗*p* < 0.05, ∗∗*p* < 0.01, and ∗∗∗*p* < 0.001.

**Figure 7:**
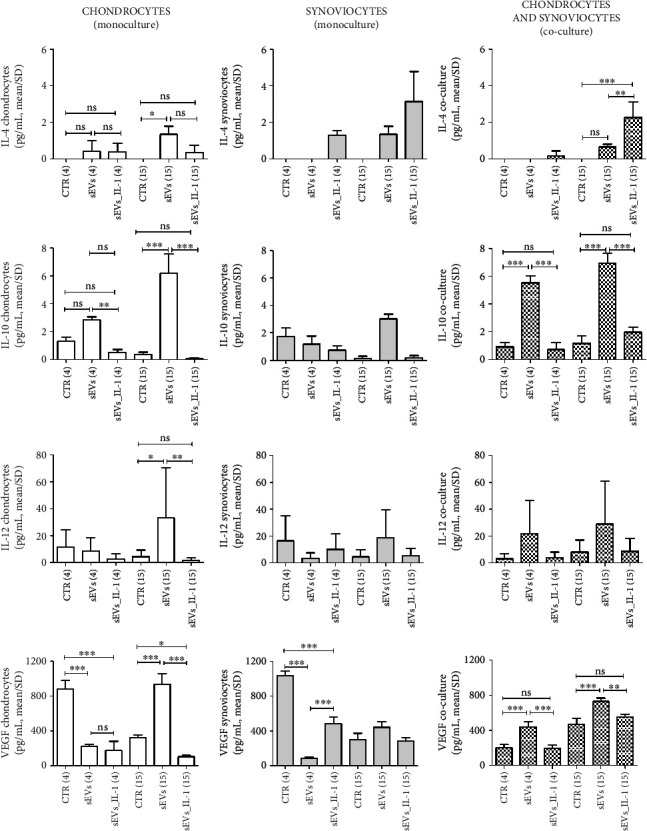
sEVs_IL-1 effects on the protein release of another subset of cytokines with reported antiinflammatory effects (IL-4, IL-10, IL-12, and VEGF) as assessed by Bioplex. Flanked results obtained from chondrocytes (white pattern), synoviocytes (grey pattern), and co-culture of these cells (pixelated pattern). For each cell type, results are shown obtained in CTR, sEVs, or sEVs_IL-1 conditions. Each graph reports data collected from 3 independent sEVs samples and expressed as means ± SD. Data were compared by ANOVA, followed by Tukey's post hoc test, with ∗*p* < 0.05, ∗∗*p* < 0.01, and ∗∗∗*p* < 0.001.

To verify the ability of sEVs_IL-1 to reproduce an OA-like environment, the protein release of a 27 plex panel of cytokines/chemokines was investigated by Bioplex technology. Supernatants obtained from both monocultures and co-cultures were evaluated. As with gene expression data, we provided the flanked results of the release from chondrocytes, synoviocytes, and co-culture, with the same scale of the *y*-axis, in order to compare the differential activities and effects (Figures 6–7 that represent the effects on different subsets of soluble factors: on major cytokines and chemokines, Figure 6 and on another subset of cytokines including growth factors, Figure 7 and Supplementary Figures 6 and 7). In addition, in each comparison at both 4 and 15 hours, the values obtained with unstimulated sEVs were shown, to tease out the specific effects of sEVs_IL-1.

In monocultures, sEVs_IL-1*β* confirmed their differential effects depending on the experimental time scheduled. At 4 h on chondrocytes, levels of IL-1ra, IL-8, and GM-CSF were significantly higher in sEVs_IL-1 with respect to CTR, while VEGF was downregulated by sEVs_IL-1 compared to CTR. At 15 h instead, sEVs_IL-1 induced a significantly higher release of IL-1*β*, IL-6, IL-8 (Figure 6), RANTES and IP-10 (Supplementary Figure 6), G-CSF, and GM-CSF (Supplementary Figure 7), with respect to the CTR, while VEGF was downregulated by sEVs_IL-1 compared to CTR (Figure 7).

On synoviocytes, sEVs_IL-1 induced a significantly higher production of IL-1*β*, IL-1ra, TNF-*α*, IL-6, IFN-*γ*, IL-8, and MCP-1 (Figure 6) as well as of IL-9 and bFGF (Supplementary Figure 7) at both 4 and 15 h. MIP-1*α*, RANTES, eotaxin, IP-10 (Supplementary Figure 6), and GM-CSF were significantly higher compared to the control only at 15 h. MIP-1*β* was significantly higher in sEVs_IL-1 group only at 4 h (Supplementary Figure 6).

The analysis of supernatants derived from the co-cultures overall indicated a more penetrant effect of the release from the synoviocytes, since in most cases, the extent of the release across the different conditions paralleled that of synoviocytes cultured in monoculture. Following sEVs_IL-1 exposure, a significantly increased release was observed at both 4 and 15 h for IL-1*β*, TNF-*α*, IFN-*γ*, IL-8, and MCP-1 (Figure 6).

### 3.4. sEVs_IL-1 Effects on p65

**Figure 8:**
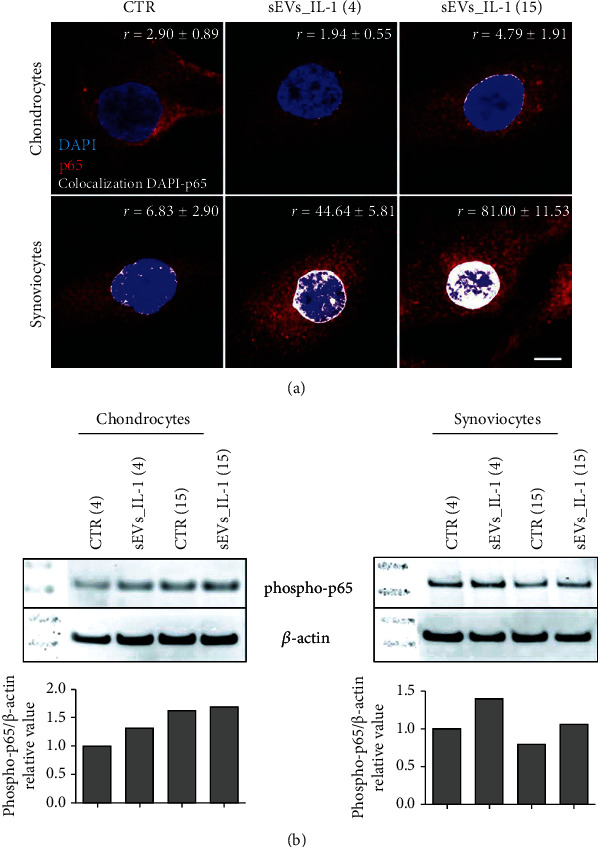
sEVs_IL-1 effects on NF-*κ*B activation. (a) p65 immunofluorescence on chondrocytes (upper row) and synoviocytes (lower row) in control conditions (CTR) and at 4 and 15 h after exposure to sEVs_IL-1. Scale bar: 10 *μ*m. Each image reports the level of the colocalization of the red (p65) to the blue (DNA) signal. The colocalization of the fluorochromes (shown in white) was quantified using Pearson's colocalization coefficient (*r*), derived from 15 analyzed optical sections and expressed as percentage ± SD. 5 cells were considered for each condition. (b) Western blot analysis of samples of chondrocytes (left) and synoviocytes (right) in CTR or sEVs_IL-1 condition at both 4 hr and 15 hr post-delivery. These samples were dedicated to western blot analysis of phosphorylation of p65 and *β*-actin as loading control. Lower graphs: relative quantification of phospho-p65 signal normalized to that of *β*-actin.

To explore the extent at which sEVs_IL-1 can activate the NF-*κ*B signaling pathway, the translocation of RelA (p65) from the cytoplasm to the nucleus was investigated in both cell types. The analysis of colocalization of the p65 (red) to the DNA (blue) signal, suggestive of p65 binding to chromatin and therefore NF-*κ*B-dependent transcription, pointed at a much stronger NF-*κ*B activation in synoviocytes. In chondrocytes, there was a prevalent cytoplasmic accumulation of p65 in both CTR and sEVs_IL-1 group, with a partial p65 localization in the nucleus only at 15 h (Figure 8(a)). Conversely, in synoviocytes, p65 was prevalently localized into the nuclei of sEVs_IL-1 treated cells, tightly associated to the DNA. This was already evident at 4 hours and nearly doubled at 15 h.

**Figure 9:**
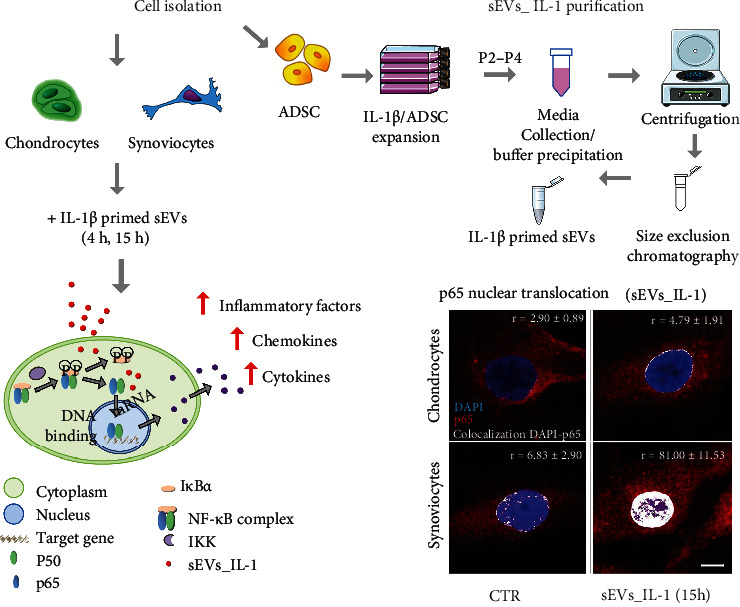
Experimental outline of the research work undertaken in the paper: sEVs_IL-1 collection from IL-1 treated ADSC; purification of sEVs_IL-1 through a combined precipitation and size exclusion chromatography method; addition of sEVs_IL-1 to primary cultures of chondrocytes and synoviocytes; analysis of the effects at the gene and protein level and molecular hypothesis underlying the observed effects.

Western blot analysis of parallel samples confirmed a more evident increased phosphorylation of p65 at both 4 and 15 hours post-sEVs_IL-1 treatment in synoviocytes compared to chondrocytes (Figure 8(b)). These findings, along with the overall experimental design, the main results of the study, and the underlying molecular mechanisms, are shown in Figure 9. Collectively, the data presented in the manuscript indicate that sEVs_IL-1 are able to transfer an inflammatory stimulus to joint cells. This is also confirmed by a similar level of expression of major inflammatory genes such as IL-8 and IL-6 in both chondrocytes and synoviocytes, either after IL-1*β* stimulation or after exposure to sEVs_IL-1 (Supplementary Figure 8). The inflammatory status of ADSC after a prolonged exposure to IL-1*β* is also in keeping with triggering of cell senescence (Supplementary Figure 2).

## 4. Discussion

The main findings of this study combined with those described in [5] point at the dual potential of ADSC secretome, which can be significantly affected by the inflammatory environment.

The primary cells (ADSC, chondrocytes, and synoviocytes) used in [5] and in the present manuscript are the same, but exposed to differential preconditioning. Therefore, combining the findings of the two manuscripts, we can emphasize that indeed ADSC-derived sEVs have a dual potential, since sEVs from control ADSC (sEVs) are able to reduce the inflammatory/catabolic environment in joint cells previously treated with IL-1*β*, and conversely sEVs from IL-1*β* primed ADSC (sEVs_IL-1) are able to induce inflammation in unstimulated cells. The data in support of this “dual potential” derive from experimental settings where the same ADSC received a differential *in vitro* preconditioning. In the present paper, this preconditioning is represented by IL-1*β* exposure, to mimic the behavior of stromal cells derived from adipose tissue of patients with MetS.

The readout was represented by an extensive analysis of the effects at the RNA and protein level on primary cultures of chondrocytes and synoviocytes in either monoculture or co-culture. In fact, sEVs_IL-1 were able to transfer NF-*κ*B activation to chondrocytes and synoviocytes, markedly enhancing the transcription and release of major inflammatory chemokines and cytokines, as well as the transcription of major cartilage catabolic enzymes. This activity was peculiar of sEVs_IL-1, since control sEVs elicited in most cases nonsignificantly different effects compared to control conditions. This is of major clinical relevance, as most OA patients are overweight or even obese.

Adipose tissue inflammatory changes with obesity and their effects at systemic level have been long recognized. WAT plays an endocrine role in MetS: M1 macrophages and ADSC release inflammatory cytokines, chemokines, and adipokines [8]; adipocytes and ADSC [37] release dysregulated circulating miR [38]. Among their putative targets, there are proteins which may afford protection in conditions of metabolic or oxidative stress including AMPK, PPAR*γ*, and PKC*ε* [38]. Their dysfunction might therefore result in increased DNA damage and senescence in ADSC, short-circuiting their stemness [37], and post-mitotic tissues (i.e., cartilage) disrupting the existing cellular homeostasis [39, 40]. This study underlined how these effects also translated in unwanted negative effects when sEVs_IL-1 (sEVs from ADSC “inflammatory” primed secretome) were applied to cells of the two main articular tissues affected by OA: chondrocytes and synoviocytes.

The robustness of the synoviocyte response is of particular interest: After exposure to sEVs_IL-1, a clear evidence of a prompt NF-*κ*B activation was appreciable by imaging these cells, largely exceeding that of chondrocytes. This is in keeping with the different kinetics of sEVs_IL-1 uptake in the two different cell types [5] and is mirrored by an overall more penetrant induction of inflammatory-catabolic markers in synoviocytes, particularly at the level of secreted molecules: major proinflammatory cytokines and chemokines.

A major mechanism of sEVs uptake is the binding of sEVs CD44 to the pericellular hyaluronan (HA) that is particularly abundant on the synoviocytes [33] either attached to hyaluronan synthase localized on cell surface or bound to plasmalemmal CD44. Hyaluronan is also highly expressed on the chondrocyte surface (pericellular matrix, PCM) as well at the level of interterritorial matrix (ICM). Yet, in chondrocytes, CD44-bound HA also serves for the organization of PCM, namely, for anchoring aggrecan to form the so-called pericellular glycocalyx, so that the interaction between sEVs CD44 and surface HA in chondrocytes is somehow hindered [41], and sEVs uptake is delayed compared to synoviocytes [5]. Furthermore, the comparison of the surface characterization of sEVs_IL-1 with that reported in [5] indicates a higher (though not statistically significant different) expression of CD44 that is also the surface molecule with the highest expression in sEVs_IL-1, thus supporting the highest activity of the latter on synoviocytes compared to chondrocytes.

At the level of gene transcription, a strong sEVs_IL-1-dependent enhancement was evident particularly at the short time exposure (4 h), while at 15 h, in most cases, a reduced gene expression was observed compared to 4 h, possibly due to the presence of instability elements in transcripts for inflammatory molecules [35]. On the other hand, at the level of release, most factors remained strongly increased at 15 h. This is expected, since cells continue to secrete factors that add on the levels at earlier time points. Some factors showed a delayed release, such as IP-10 that in chondrocytes was almost undetectable at 4 h while detected at high concentration at 15 h. The effects of sustained release can be evidenced, particularly for synoviocytes (IL-1*β*, IL-1ra, TNF*α*, IL-6, GM-CSF, IFN-*γ*, MIP-1*α*, RANTES, IP-10 and eotaxin).

Gene expression of major OA cartilage catabolic enzymes was also significantly increased by sEVs_IL-1 in monoculture and co-cultured chondrocytes: aggrecanase ADAMTS-4, a biomarker for joint inflammation and effusion [42], and a first line enzyme in OA pathogenesis, responsible for aggrecan removal and exposure of collagen 2 to the activity of major collagenases: MMP-1 and particularly MMP-13, a pivotal enzyme in driving chondrocyte towards hypertrophy and terminal differentiation [43]. Collagenases need a proteolytical activation that may occur thanks to the activity of MMP-10 [44] that is also increased in chondrocytes exposed to sEVs_IL-1. Expression of major collagenases (MMP-1 and MMP-13) and ADAMTS-4 was significantly induced in monoculture synoviocytes, while expression of MMP-1, -13, and ADAMTS-4 was also significantly increased in co-cultured synoviocytes, confirming that in this culture model, autocrine and paracrine loops may further enhance the catabolic environment. Of particular interest is the strong induction of ADAMTS-4 that is typically dependent on inflammatory stimuli [45], unlike ADAMTS-5.

Along with the effects on the release of known inflammatory and catabolic factors, noteworthy activity was also observed on a group of cytokines that were previously reported as having protective effects on OA cells. Classically, the chondroprotective cytokines are IL-4, IL-10, and IL-13 [46]. Of these, only IL-4 and IL-10 were detected in the supernatant, while IL-13 was below the detection limit. Compared to the control sEVs, IL-10 release was significantly downregulated by sEVs_IL-1 at the early time point, in both chondrocytes and co-cultures. Interestingly, a similar trend was observed for IL-12 and VEGF in co-cultures. VEGF release in particular is significantly downregulated by sEVs_IL-1 compared to the control conditions in both chondrocytes and synoviocytes. VEGF has been associated with OA worsening for the pathological consequences of neoangiogenesis in both cartilage and synovium. However, VEGF signaling is important to keep a healthy WAT, preventing its expansion, the whitening of brown adipose tissue, and a decreased energy consumption [47], thus ultimately preventing the worsening of the MetS. Interestingly, the kinetic trend of these cytokines was opposite compared to that shown in [5], further confirming that opposite mechanisms are triggered by sEVs derived from ADSC exposed or not to IL-1*β*.

Given the potential of sEVs, major efforts should be devoted in developing techniques whereby assessing and engineering the type of carried information and eventually in scaling up the production of these “nanodrugs” exploiting bioreactors to ensure reproducibility or even exploring the prospective of allogeneic or xenogeneic production [48]. Indeed, current evidence suggests the requirement of a careful characterization of ADSC before embarking in secretome collection for therapeutic purposes. In fact, circulating sEVs from patients with MetS are enriched in lipopolysaccharide and may trigger toll-like receptor 4 (TLR4) [49] in the endothelium, thus enhancing cytosolic and mitochondrial oxidative stress. TLR4 is also abundant in cartilage, therefore similar mechanisms may concur in driving MetS OA [50]. The dysfunctionality of MSC and sEVs obtained from obese subjects has also been confirmed in obese mice [18, 51, 52] where ADSC show increased level of senescence and a lower proliferation. In particular, the activation of the IL-1 pathway has been confirmed by means of proteomic and transcriptomic evaluation in ADSC of mice treated with high fat diet, and this further confirms that the present study may mimic the condition of ADSC derived from obese patients. Similar findings were obtained in pigs in a study where a comparison was undertaken between ADSC derived from lean pigs or pigs fed a high fat diet. ADSC from obese animals show an increased senescence and NF-*κ*B activation [53]. In addition, miRnome and transcriptome analysis of ADSC from lean and obese pigs confirmed that the differences were focused to proteins involved in transcription, mitochondria, and inflammation [53].

This study presents some limitations. First, the sample size is limited, yet sufficient to disclose significant effects. Second, the issue of the sEVs is still under definition, and the subset of vesicles we purified span over a size range (30-202 nm, with 60% vesicles sized 50-100 nm [5]) where a miscellaneous of different vesicles could co-purify: exosomes, exomeres, microvesicles, ectosomes, or microparticles [54]. Third, despite the clear-cut effects, a thorough molecular understanding of the mechanisms (sEVs mediated transfer of mRNA, miRNA, or other short RNAs active in posttranscriptional regulation) whereby NF-*κ*B tuning occurs is still awaited.

However, the study findings were also able to point out the dual nature of adipose tissue according to its inflammatory state. While preliminary clinical findings show promise toward favorable clinical effects, the potential of adipose derived treatments may be negatively affected by the inflammatory changes linked to the MetS. This warrants more attention toward better characterizing the most suitable adipose products and identifying the patient profile which might more likely benefit from this biological approach to address OA. This implies that prior of their use in regenerative medicine, further efforts may be dedicated at characterizing the extent of ADSC senescence as previously mentioned [55, 56]. This “senescence fingerprinting” of ADSC would allow to select the ADSC with true homeostatic properties and avoid the use of senescent cells that could worsen OA [57]. Moreover, given the cell-free nature of a sEVs based therapy, this could lend itself to a bioreactor-based scaled up production, thus even envisioning allogeneic strategies. At the same time, the power of *in vitro* preconditioning may be fully exploited to rescue/enhance the protective/homeostatic nature of ADSC released sEVs via the use of senotherapeutic (senolytic/senomorphic) molecules [58].

## 5. Conclusions

These study findings warrant caution before using ADSC for secretome collection and OA treatment. A careful assessment of the inflammatory commitment of these cells should be undertaken, to avoid worsening of OA via transfer of NF-*κ*B activation to joint cells. At the same time, the current state of the art suggests that the immunomodulatory activity of ADSC and their secretome may be rescued *in vitro.* Therefore, a promising perspective is to focus on the workout of techniques whereby obtaining large and qualitatively reproducible amounts of sEVs from batches of allogeneic/xenogeneic ADSC through refinement of several enhancing strategies that span from *in vitro* culture models to epigenetic manipulation, toward the development of a more effective OA treatment.

## Figures and Tables

**Table 1:** List of primers used in real-time PCR.

RNA template	Primer sequences (forward:5′ and reverse:3′)	Annealing temperature (°C)
GAPDH	5′-TGGTATCGTGGAAGGACTCATGAC 3′-ATGCCAGTGAGCTTCCCGTTCAGC	60
IL-1*β*	5′-GTGGCAATGAGGATGACTTGTT 3′-TGGTGGTCGGAGATTCGTAG	60
IL-6	5′-TAGTGAGGAACAAGCCAGAG 3′-GCGCAGAATGAGATGAGTTG	60
IL-8	5′-CCAAACCTTTCCACCC 3′-ACTTCTCCACAACCCT	60
MCP-1	5′-GAAGCTCGCACTCTCGCCT′ 3′-GAGTGTTCAAGTCTTCGGA′	60
TNF-*α*	5′-AGCCCATGTTGTAGCAAACC 3′-ACCTGGGAGTAGATGAGGTA	60
MMP-1	5′-TGGACCTGGAGGAAATCTTG 3′-CCGCAACACGATGTAAGTTG	60
MMP-10	5′-GCCAGTCCATGGAGCAAGGCT′ 3′-TCGCCTAGCAATGTAACCAGCTGT	60
ADAMTS4	5′-CTGCCTACAACCACCG 3′-GCAACCAGAACCGTCC	60
ADAMTS5	5′-GCACTTCAGCCACCATCAC 3′-AGGCGAGCACAGACATCC	60
MMP-13	5′-TCACGATGGCATTGCT 3′-GCCGGTGTAGGTGTAGA	60
COX-2	5′-CAGCACTTCACGCATCAGTTT 3′-GCGCAGTTTACGCTGTCTA	60
INOS	5′-ACATTGATCAGAAGCTGTCCCAC 3′-AAAGGCTGTGAGTCCTGCAC	60
IL-10	5′-CTTTAAGGGTTACCTGGGTTG 5′-CTTGATGTCTGGGTCTTGG	60
VEGF	5′-TGATGATTCTGCCCTCCTC 3′-GCCTTGCCTTGCTGCTC	60

## Data Availability

The data reported in this study are available upon reasonable request from the corresponding author.

## Supplementary Materials

Supplementary Materials S1: surface epitope characterization of small extracellular vesicles and comparison of the level of expression among the different markers. S2: IL-1*β* exposure induces cell senescence in ADSC as evidenced by different markers. S3: stability test of GAPDH in the experimental setting described in [5] and in the present manuscript. S4: sEVs_IL-1 effects on gene expression in chondrocytes. S5: sEVs_IL-1 effects on gene expression in synoviocytes. S6: sEVs_IL-1 effects on the release of chemokines. S7: sEVs_IL-1 effects on the release of cytokines. S8: flanked results at the gene expression level obtained from the same cells after different preconditioning of both ADSC and joint cells (chondrocytes or synoviocytes).
