# Supplementary material for: Small Extracellular Vesicles from Inflamed Adipose Derived Stromal Cells Enhance the NF-κB-Dependent Inflammatory/Catabolic Environment of Osteoarthritis
Source: Stem Cells Int. 2022 Jul 18;2022:9376338. doi: 10.1155/2022/9376338 (PMC9314187; doi:10.1155/2022/9376338)
Supplement: Supplementary Materials — S1: surface epitope characterization of small extracellular vesicles and comparison of the level of expression among the different markers. S2: IL-1β exposure induces cell senescence in ADSC as evidenced by different markers. S3: stability test of GAPDH in the experimental setting described in [5] and in the present manuscript. S4: sEVs_IL-1 effects on gene expression in chondrocytes. S5: sEVs_IL-1 effects on gene expression in synoviocytes. S6: sEVs_IL-1 effects on the release of chemokines. S7: sEVs_IL-1 effects on the release of cytokines. S8: flanked results at the gene expression level obtained from the same cells after different preconditioning of both ADSC and joint cells (chondrocytes or synoviocytes). [file 9376338.f1.pdf]

SUPPLEMENTARY MATERIAL

Small Extracellular Vesicles from Inflamed Adipose Derived Stromal Cells Enhance the NF-κB Dependent Inflammatory/Catabolic Environment of Osteoarthritis

Carola Cavallo[1], Giulia Merli[2], Nicoletta Zini[3,4], Stefania D'Adamo[5,6], Luca Cattini[6], Michele Guescini[7], Brunella Grigolo[1], Alessandro Di Martino[8], Spartaco Santi [3,4], Rosa Maria Borzì[6,#], and Giuseppe Filardo[2,#]

[1] Laboratorio RAMSES, IRCCS Istituto Ortopedico Rizzoli, Bologna, 40136, Italy.

[2] Applied and Translational Research center (ATRc), IRCCS Istituto Ortopedico Rizzoli, Bologna, 40136, Italy.

[3] CNR Institute of Molecular Genetics "Luigi Luca Cavalli-Sforza", Unit of Bologna, Bologna, 40136, Italy.

[4] IRCCS Istituto Ortopedico Rizzoli, Bologna, 40136, Italy.

[5] Department of Medical and Surgical Sciences, University of Bologna, Bologna, 40138, Italy.

[6] Laboratorio di Immunoreumatologia e Rigenerazione Tissutale, IRCCS Istituto Ortopedico Rizzoli, Bologna, 40136, Italy

[7] Department of Biomolecular Science, University of Urbino Carlo Bo, Urbino, 61029, Italy.

[8] Clinica Ortopedica e Traumatologica 2, IRCCS Istituto Ortopedico Rizzoli, Bologna, 40136, Italy.

# These authors contributed equally to this work

Supplementary table 1. Information (age, sex and BMI) about the patients yielding the ADSC, the chondrocyte and the synoviocyte cultures used in [1] and in the present manuscript.

CELL TYPE	AGE	SEX	BMI	HEIGHT	WEIGHT
**ADSC**	**AGE**	**SEX**	**BMI**	**Height (cm)**	**Weight (Kg)**
#1	35	M	26	190	94
#2	70	M	28.41	178	90
#3	69	F	43.71	165	119
**Chondrocytes**	**AGE**	**SEX**	**BMI**	**Height (cm)**	**Weight (Kg)**
#1	75	F	24.17	164	65
#2	58	M	24.34	172	72
#3	60	F	25.64	158	64
**Synoviocytes**	**AGE**	**SEX**	**BMI**	**Height (cm)**	**Weight (Kg)**
#1	69	M	32.88	177	103
#2	69	M	29.07	171	95

Supplementary table 2. Experimental settings underlying the study reported in [1] and in the present manuscript. In the present manuscript, for each cell type (chondrocyte pool and synoviocyte pool) in either monoculture or co-culture and kept in control conditions we undertook an evaluation of the effects of sEVs from ADSC exposed to IL-1β (sEVs_IL-1) in comparison with the effects of sEVs from control ADSC (sEVs) at both 4 and 15 hours. Data obtained in this work were also compared to the results of our previous study investigating on both chondrocytes and synoviocytes prior exposed to IL-1β the effects of sEVs from ADSC (sEVs) [1].

**present manuscript**						**Cavallo et al, Scientific Reports 2021[1]**			
**CTR(4)**	**sEVs(4)**	**sEVs_IL-1(4)**	**CTR(15)**	**sEVs(15)**	**sEVs_IL-1(15)**	**CTR_IL-1(4)**	**sEVs(4)**	**CTR_IL-1(15)**	**sEVs(15)**

S1 (supplementary figure 1)

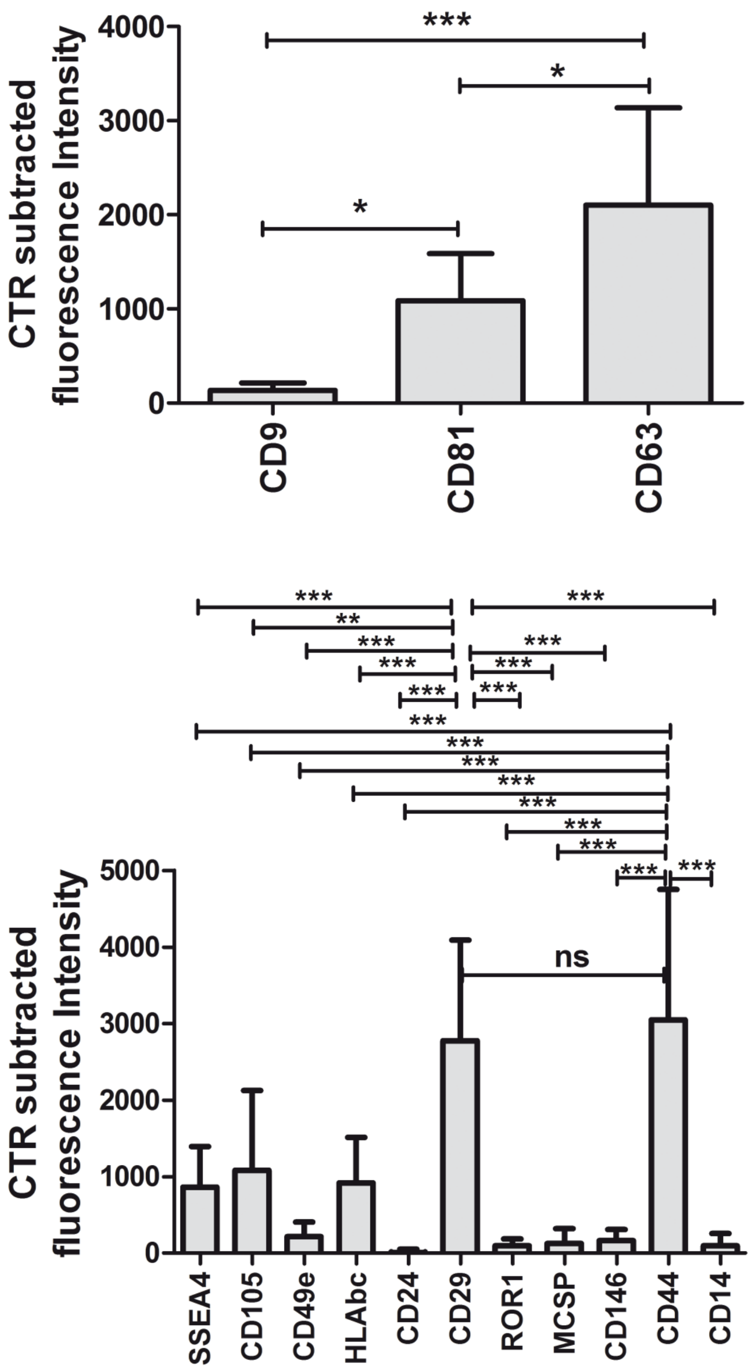

S2 (supplementary figure 2)

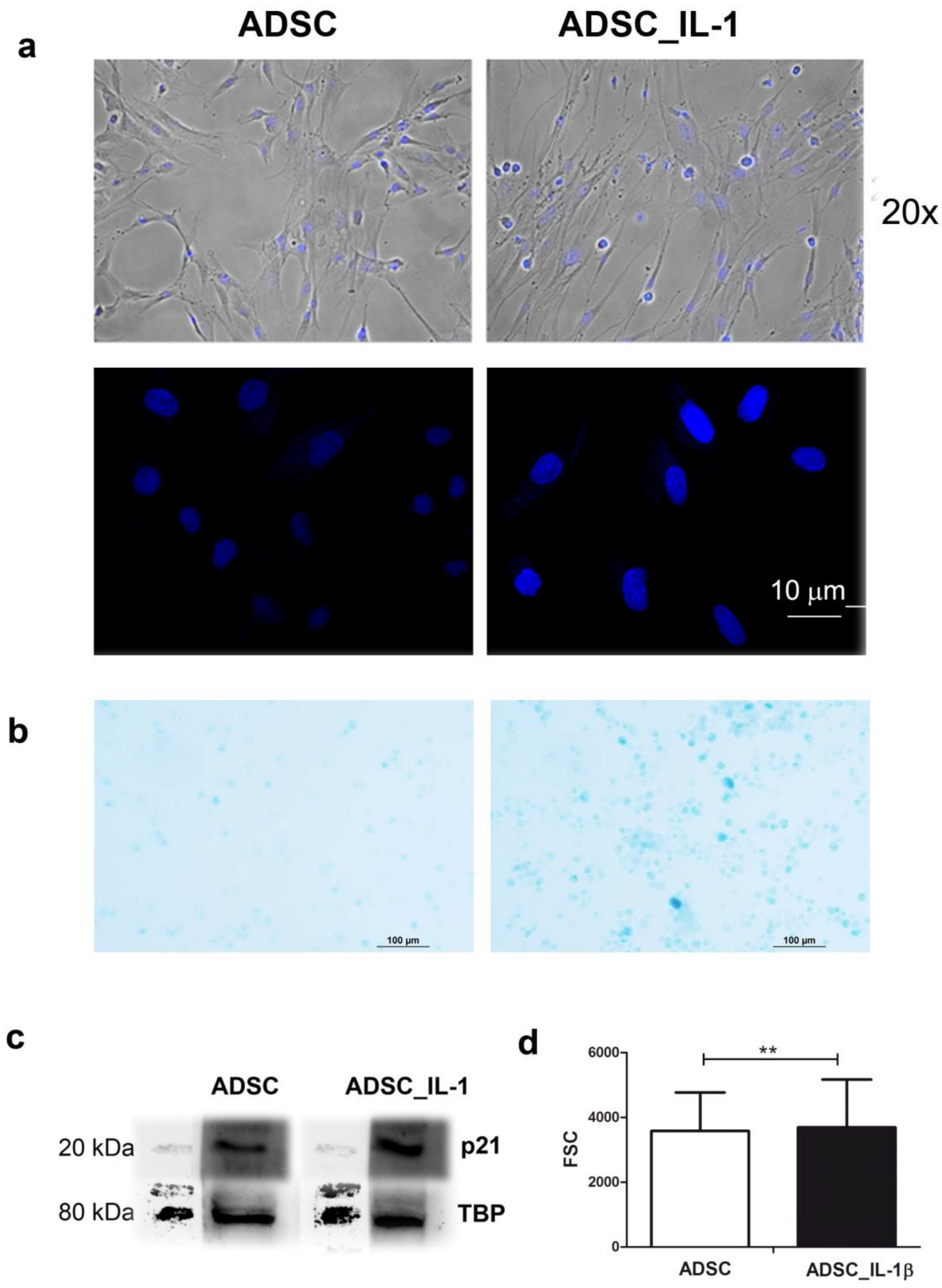

S3 (supplementary figure 3)

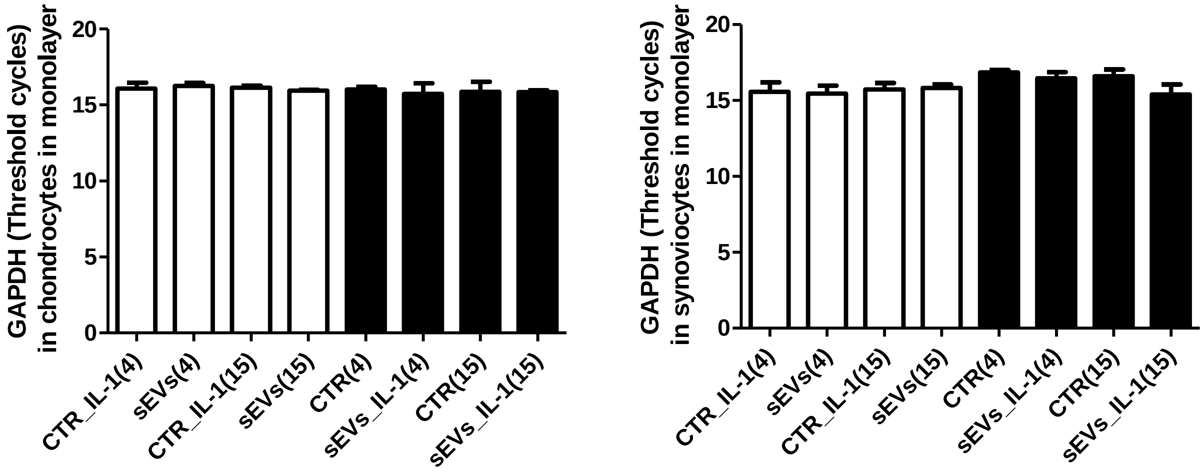

S4 (supplementary figure 4)

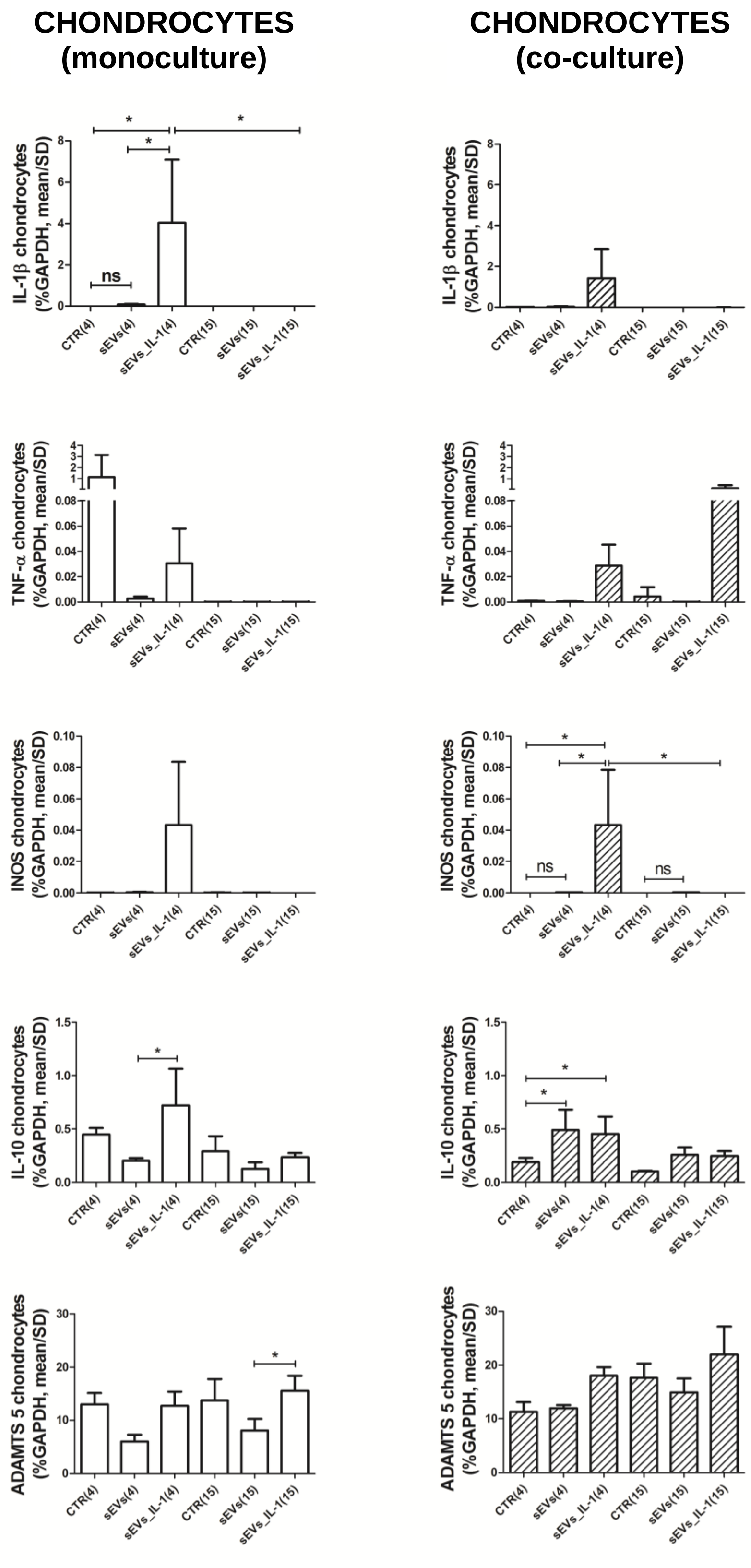

S5 (supplementary figure 5)

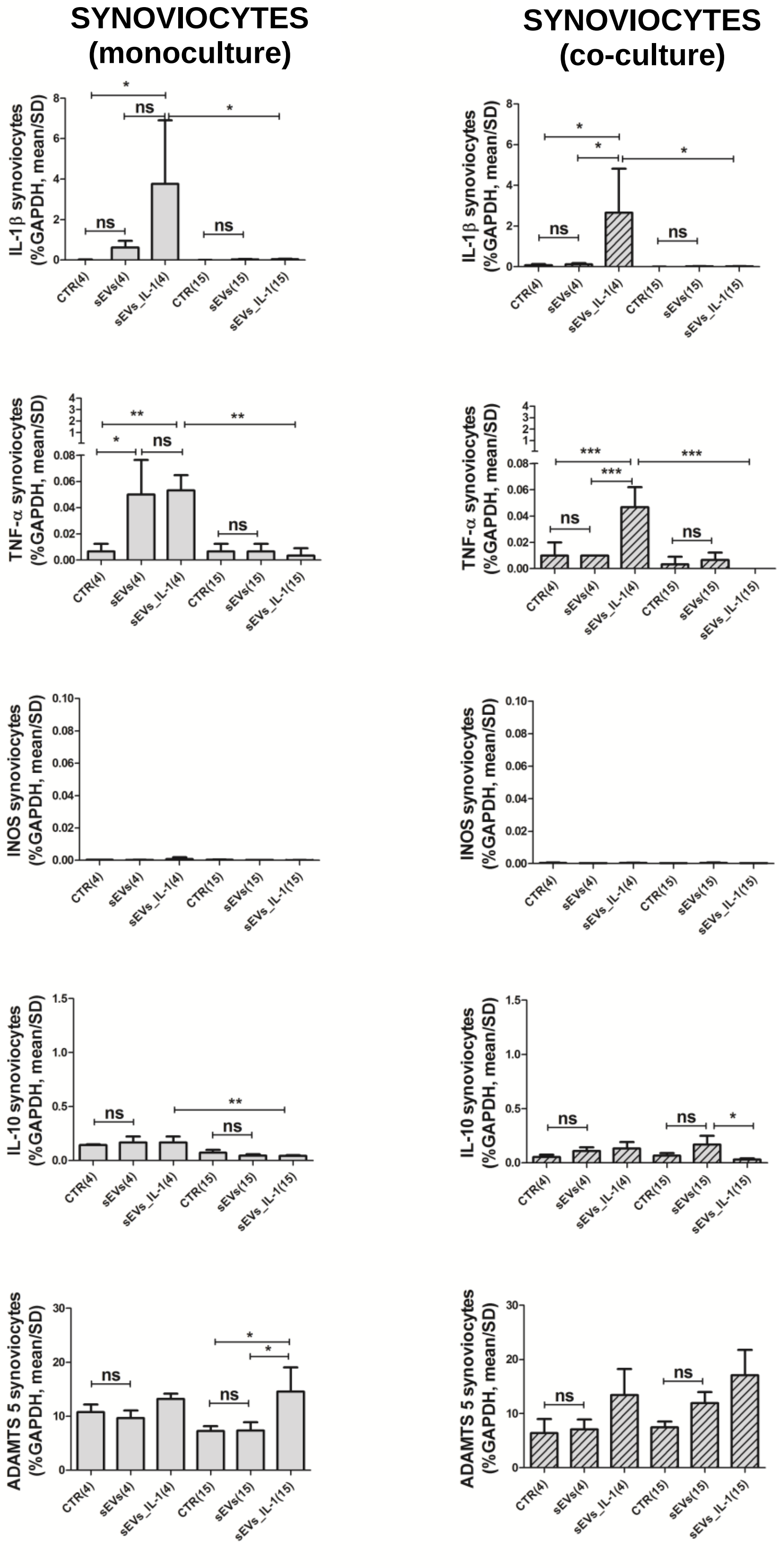

S6 (supplementary figure 6)

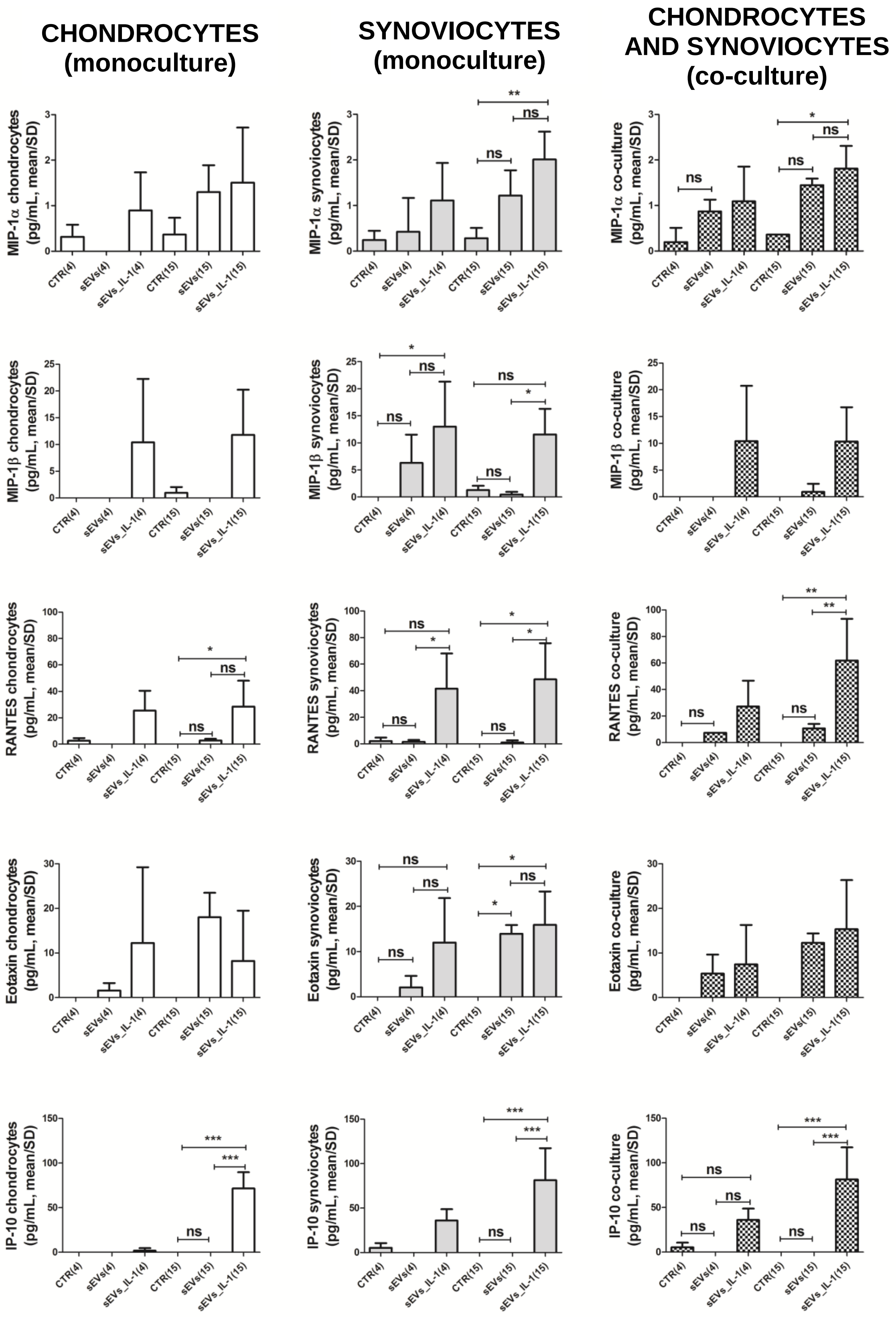

S7 (supplementary figure 7)

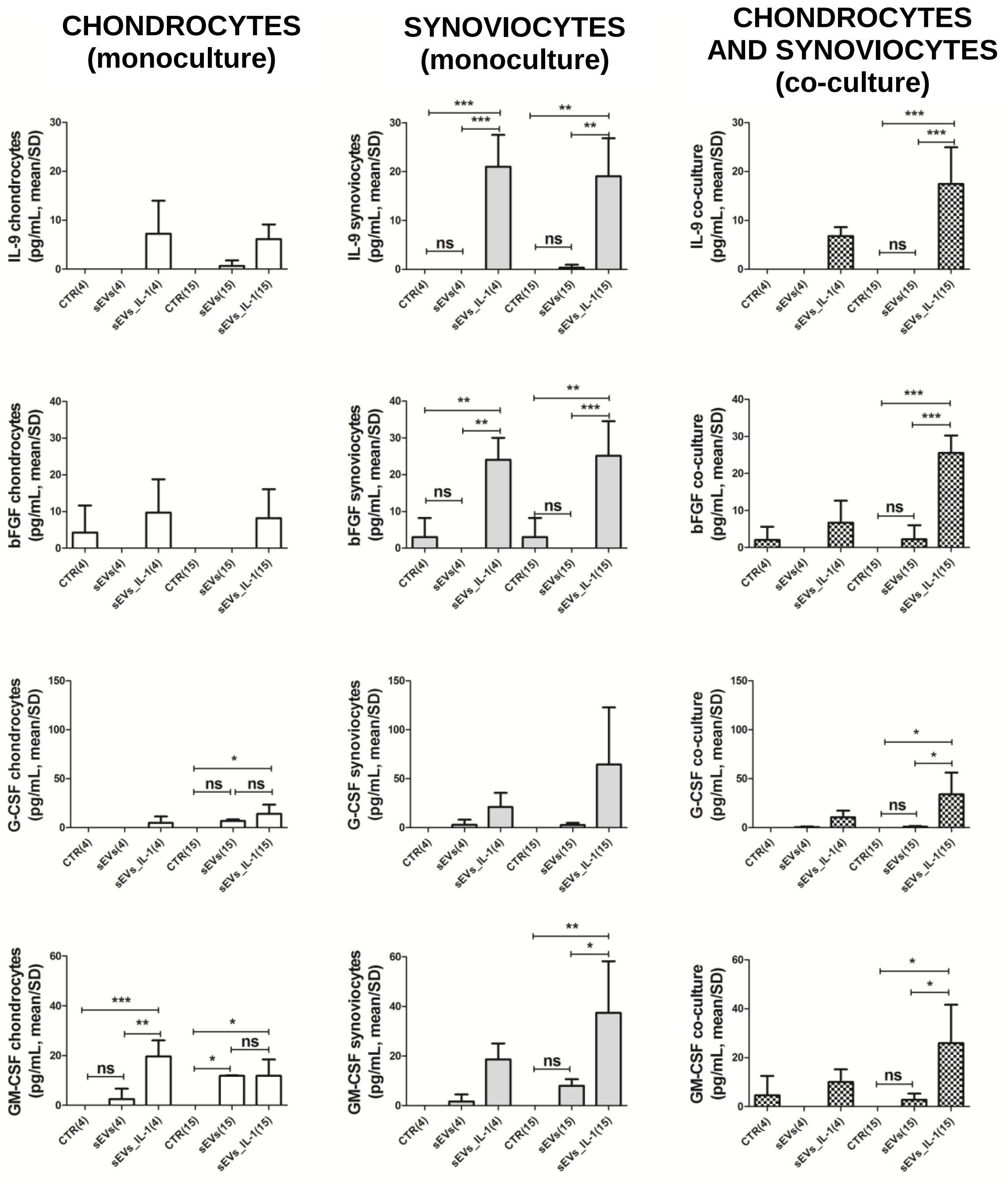

S8 (supplementary figure 8)

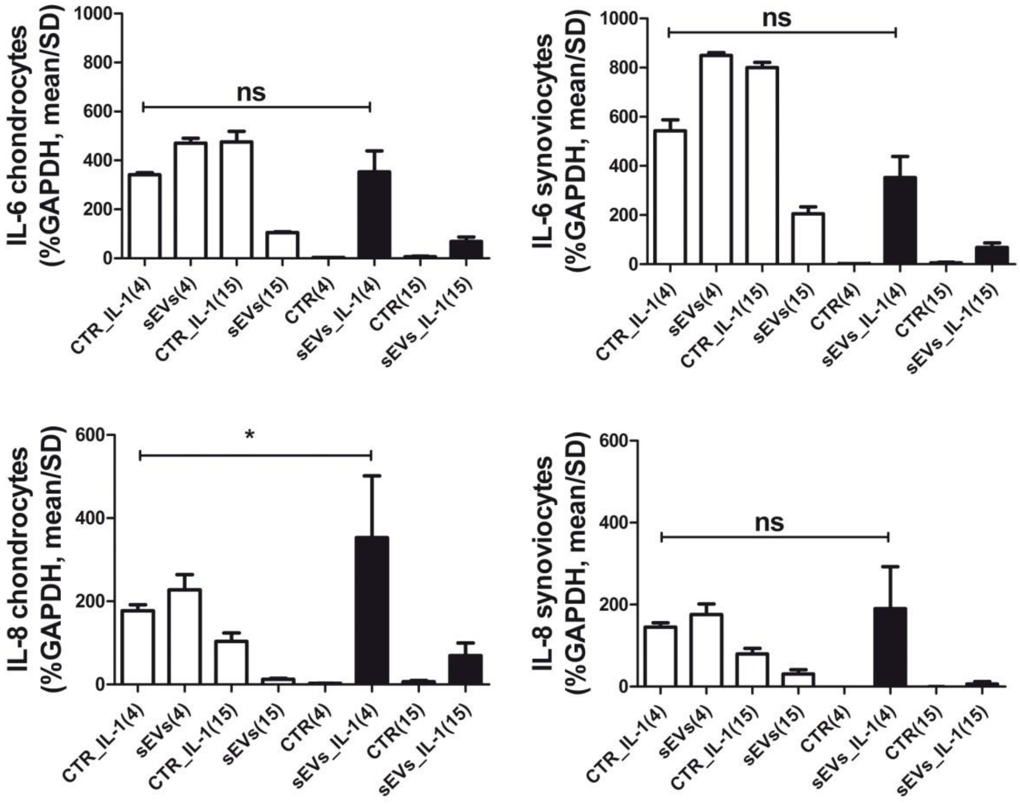

LEGENDS OF SUPPLEMENTARY FIGURES

Supplementary Figure 1. Surface Epitope characterization of Small Extracellular Vesicles. As detailed in the main manuscript, sEVs and sEVS_IL-1 (n=3, each) underwent whole MACSPlex Exosome Kit surface assessment of 37 surface markers and 2 isotype controls and the data were expressed as net fluorescence intensity (control subtracted fluorescence intensity) and displayed as mean ± SD. To obtain a kind of comparison of the different level of expression among the markers, the data of sEVs and sEVs_IL-1 were cumulated and the level of expression of the different markers were compared by mean of ANOVA with Tukey's post hoc test (paired data). The results are shown in two different graphs. Upper graph: comparison of the fluorescence intensity of the tetraspanins within the cumulative data (including both sEVs and sEVs_IL-1, with * $p<0.05$ and *** $p<0.001$). Lower graph: comparison of the fluorescence intensity of the other markers: CD44 and CD29 show the highest level of expression, there is no statistical significant difference between CD44 and CD29, while both are expressed at statistically significant higher level compared to all the other markers (*** $p<0.001$ in comparison to each other marker for both CD29 and CD44, but CD105 that was significantly lower compared to CD29 with **$p< 0.01$).

Supplementary Figure 2. IL-1β exposure induces cell senescence in ADSC as evidenced by different markers. (a) Long term IL-1β treatment of ADSC induced marked modification of cell shape in a relevant fraction of cells that appeared round without body extensions (first row, 20x magnification of fields with overlay of images taken with dichroic filters with images with DAPI nuclear counterstaining). Nuclear counterstaining points at the presence of condensed chromatin in the cells after prolonged exposure to IL-1β (second row, images of the nuclei captured by confocal laser scanning microscopy), suggestive of attempted repair of DNA damage[2]. (b) Induction of cell senescence is further confirmed by higher SA-β Gal activity detected in cytospin of ADSC_IL-1 in comparison with control ADSC, processed as detailed in [3]. (c) western blot of p21 (with TATA binding protein as a loading control) of the same cell equivalents (150,000 cells) shows increased p21 expression in ADSC_IL-1: left, ADSC cultured in control condition as described in [1] and right, ADSC exposed to IL-1β. (d) ADSC_IL-1 cells show higher cell size as detected by flow cytometry analysis of forward size scatter (mean ± standard deviation of nearly 2,500 cells, ** $p<0.01$). Increased cell size is another feature of senescence as detailed in [4].

Supplementary Figure 3. Stability test of GAPDH in the experimental settings described in [1] and in the present manuscript. The figure reports the mean±standard deviation of the threshold cycles for the GAPDH gene used as a reference control, in both chondrocytes (left figure) and synoviocytes (right figure). Each condition refers to n=3. These values were obtained with equal amounts of cDNAs obtained with 200 ng input RNA each. White pattern is relative to the data presented in [1], while the black pattern is relative to the data presented in the current manuscript.

ANOVA analysis confirmed no statistical significance among the data and therefore the stability of GAPDH as a reference gene, thus confirming that GAPDH is a suitable gene to compare gene expression among the various conditions presented in this manuscript.

Supplementary Figure 4. IL-1β sEVs effects on gene expression in chondrocytes, in both monostrate culture (white pattern) or in co-culture (dashed pattern). These data complement those shown in Figure 2 and 3 of the main manuscript. Comparisons among multiple groups (CTR, control sEVs or IL-1β sEVs-treated cells at 4 and 15 hours) were carried out with ANOVA, followed by Tukey's post hoc test. The differences were considered significant and evidenced as *$p < 0.05$.

Supplementary Figure 5. IL-1β sEVs effects on gene expression in synoviocytes, in both monostrate culture (grey pattern) or in co-culture (dashed pattern). These data complement those shown in Figure 4 and 5 of the main manuscript. Comparisons among multiple groups (CTR, control sEVs or IL-1β sEVs-treated cells at 4 and 15 hours) were carried out with ANOVA, followed by Tukey's post hoc test. The differences were considered significant and evidenced as *p < 0.05; **p < 0.01; and ***p < 0.001.

Supplementary Figure 6. IL-1β sEVs effects on the release of chemokines, complementing the data shown in Figure 6 of the main manuscript. The figure reports data from chondrocytes (white pattern), synoviocytes (grey pattern) and coculture of these cells (pixelated pattern). These data complement those shown in Figure 6 and 7 of the main manuscript and are relative to the chemokines MIP-1α, MIP-1β, RANTES, Eotaxin and IP-10. Comparisons among multiple groups (CTR, control sEVs or IL-1β sEVs-treated cells at 4 and 15 hours) were carried out with ANOVA, followed by Tukey's post hoc test. The differences were considered significant and evidenced as *p < 0.05; **p < 0.01; and ***p < 0.001.

Supplementary Figure 7. IL-1β sEVs effects on the release of cytokines, complementing the data shown in Figure 6 and 7 of the main manuscript. The figure reports data from chondrocytes (white pattern), synoviocytes (grey pattern) and coculture of these cells (pixelated pattern). These data complement those shown in Figure 6 and 7 of the main manuscript and are relative to the cytokines IL-9, basic FGF, G-CSF and GM-CSF. Comparisons among multiple groups (CTR, control sEVs or IL-1β sEVs-treated cells at 4 and 15 hours) were carried out with ANOVA, followed by Tukey's post hoc test. The differences were considered significant and evidenced as *p < 0.05; **p < 0.01; and ***p < 0.001.

Supplementary Figure 8. Flanked results at the gene expression level obtained from the same cells after different preconditioning of both ADSC and joint cells (chondrocytes or synoviocytes). Further evidence that IL-1β sEVs are able to transfer inflammation to unstimulated cells as shown by the gene expression level of IL-6 and IL-8 in both chondrocytes (left figure) and synoviocytes (right figure). IL-6 in particular shows no statistical difference when comparing the condition presented in [1], i.e. chondrocytes stimulated for 15 hours with IL-1β with those described in the present manuscript, i.e. chondrocytes treated for 4 hours with sEVs derived from ADSC treated with IL-1β (2ng/ml). White pattern is relative to the data presented in [1], while the black pattern is relative to the data presented in the current manuscript.

REFERENCES

[1] C. Cavallo, G. Merli, R.M. Borzi, N. Zini, S. D'Adamo, M. Guescini, B. Grigolo, A. Di Martino, S. Santi, G. Filardo, Small Extracellular Vesicles from adipose derived stromal cells significantly attenuate in vitro the NF-kappaB dependent inflammatory/catabolic environment of osteoarthritis, Sci Rep 11(1) (2021) 1053.
[2] S.W. Criscione, Y.V. Teo, N. Neretti, The Chromatin Landscape of Cellular Senescence, Trends Genet 32(11) (2016) 751-761.
[3] S. Guidotti, M. Minguzzi, D. Platano, L. Cattini, G. Trisolino, E. Mariani, R.M. Borzi, Lithium Chloride Dependent Glycogen Synthase Kinase 3 Inactivation Links Oxidative DNA Damage, Hypertrophy and Senescence in Human Articular Chondrocytes and Reproduces Chondrocyte Phenotype of Obese Osteoarthritis Patients, PLoS One 10(11) (2015) e0143865.
[4] G.E. Neurohr, R.L. Terry, J. Lengefeld, M. Bonney, G.P. Brittingham, F. Moretto, T.P. Miettinen, L.P. Vaites, L.M. Soares, J.A. Paulo, J.W. Harper, S. Buratowski, S. Manalis, F.J. van Werven, L.J. Holt, A. Amon, Excessive Cell Growth Causes Cytoplasm Dilution And Contributes to Senescence, Cell 176(5) (2019) 1083-1097 e18.
